# A DFT investigation on the potential of beryllium oxide (Be_12_O_12_) as a nanocarrier for nucleobases

**DOI:** 10.1371/journal.pone.0313885

**Published:** 2024-11-22

**Authors:** Mahmoud A. A. Ibrahim, Maggie N. S. Hanna, Al-shimaa S. M. Rady, Peter A. Sidhom, Shaban R. M. Sayed, Mohamed A. El-Tayeb, Ahmed M. Awad, Hatem Tallima, Tamer Shoeib

**Affiliations:** 1 Computational Chemistry Laboratory, Chemistry Department, Faculty of Science, Minia University, Minia, Egypt; 2 School of Health Sciences, University of KwaZulu-Natal, Westville Campus, Durban, South Africa; 3 Department of Pharmaceutical Chemistry, Faculty of Pharmacy, Tanta University, Tanta, Egypt; 4 Department of Botany and Microbiology, College of Science, King Saud University, Riyadh, Saudi Arabia; 5 Department of Chemistry, California State University Channel Islands, Camarillo, California, United States of America; 6 Department of Chemistry, The American University in Cairo, New Cairo, Egypt; Atma Ram Sanatan Dharma College University of Delhi, INDIA

## Abstract

The study of the interactions between biomolecules and nanostructures is quite fascinating. Herein, the adsorption propensity of beryllium oxide (Be_12_O_12_) nanocarrier toward nucleobases (NBs) was investigated. In terms of DFT calculations, the adsorption tendency of Be_12_O_12_ toward NBs, including cytosine (NB-C), guanine (NB-G), adenine (NB-A), thymine (NB-T), and uracil (NB-U), was unveiled through various configurations. Geometrical, electronic, and energetic features for Be_12_O_12_, NBs, and their associated complexes were thoroughly evaluated at M06-2X/6-311+G** level of theory. The potent adsorption process within NBs∙∙∙Be_12_O_12_ complexes was noticed through favorable interaction (*E*_int_) and adsorption (*E*_ads_) energies with values up to –53.04 and –38.30 kcal/mol, respectively. Generally, a significant adsorption process was observed for all studied complexes, and the favorability followed the order: NB-C∙∙∙ > NB-G∙∙∙ > NB-A∙∙∙ > NB-T∙∙∙ > NB-U∙∙∙Be_12_O_12_ complexes. Out of all studied complexes, the most potent adsorption was found for NB-C∙∙∙Be_12_O_12_ complex within configuration A (*E*_int_ = –53.04 kcal/mol). In terms of energy decomposition, SAPT analysis revealed electrostatic (*E*_elst_) forces to be dominant within the studied adsorption process with values up to –99.88 kcal/mol. Analyzing QTAIM and NCI, attractive intermolecular interactions within the studied complexes were affirmed. From negative values of thermodynamic parameters, the nature of the considered adsorption process was revealed to be spontaneous and exothermic. Regarding density of state, IR, and Raman analyses, the occurrence of the adsorption process within NBs∙∙∙Be_12_O_12_ complexes was confirmed. Noticeable short recovery time values were observed for all studied complexes, confirming the occurrence of the desorption process. The findings provided fundamental insights into the potential application of Be_12_O_12_ nanocarrier in drug and gene delivery processes.

## Introduction

The advent of nanomaterials has blended with significant interest owing to their unique structure and physicochemical characteristics [1, 2]. Nanomaterials have pivotal features, paving the way for several advances in biomedical applications [3–8]. The bioavailability and biocompatibility of nanomaterials have facilitated the development of novel drug delivery systems (i.e., nanocarrier) [9–13]. Subsequently, various nanocarriers have been evaluated for their biomedical applications since the discovery of fullerene by Kroto et al. [14]. Among the developed nanocarriers, fullerene-like nanocarriers have been extensively investigated due to their promising features [15, 16].

Considering all forms of fullerene-like nanocarriers, metal oxide structures (M_12_O_12_, where M = Be, Mg, and Zn) have opened up a wide range of applications due to their intriguing properties. Metal oxide nanocarriers were extensively engaged in chemical and biochemical applications [17–19]. In this regard, metal oxide nanocarriers were included in chemical reactions and hydrogen storage materials [20–22]. Furthermore, metal oxide nanocarriers have gained more attention in drug and gene delivery processes owing to their surface area and adsorption capacity [23, 24]. As a metal oxide nanocarrier, beryllium oxide (Be_12_O_12_) has remarkable structural and thermal stability [25, 26]. Be_12_O_12_ is also characterized by a wide energy gap and a partly covalent Be-O bond. Substantially, Be_12_O_12_ has been engaged in the delivery process of drugs [27, 28]. Further, the performance of Be_12_O_12_ toward adsorbing anticancer and antihyperthyroidism drugs was investigated by means of DFT methods [29–32].

Nucleobases (NBs) are nitrogenous biological compounds that serve as the building blocks of nucleotides in deoxyribonucleic acids (DNA) and ribonucleic acids (RNA). Based on first principles, NBs contain purines (adenine (NB-A) and guanine (NB-G)) and pyrimidines (cytosine (NB-C), thymine (NB-T), and uracil (NB-U)). NBs are essential in different biological fields, particularly therapeutic applications [33, 34]. The prime impetus to use NBs is to improve the ways of treating cancer, heart disease, central nervous system diseases, and immunological diseases [35, 36]. Nevertheless, the limited stability and poor cell membrane penetration for NBs pose significant challenges [37]. Therefore, nanocarriers have been proposed as promising candidates for targeted NBs delivery processes [38, 39]. Accordingly, the adsorption of NBs over graphene was demonstrated [40, 41]. In this regard, the adsorption performance of (ZnO)_3_ cluster and C_24_ fullerene toward various NBs was illustrated [42, 43]. As a biosensing platform, the performance of aluminium nitride nanosheets, as well as pure and Al-doped boron nitride sheets, in the adsorption process of NBs was also investigated [44, 45].

Hence, the current study aims to demonstrate the targeted adsorption potential of beryllium oxide (Be_12_O_12_) nanocarrier toward the five nucleobases. Upon the density functional theory (DFT), structural, energetic, electronic, and thermodynamic features of NB-C∙∙∙, NB-G∙∙∙, NB-A∙∙∙, NB-T∙∙∙, and NB-U∙∙∙Be_12_O_12_ complexes were studied. In this regard, electrostatic potential (ESP), quantum theory of atoms in molecules (QTAIM), noncovalent interactions (NCI) index, and symmetry-adapted perturbation theory (SAPT) analyses were performed. Further, frontier molecular orbitals (FMOs), density of state (DOS), IR, and Raman analyses were executed for the studied NBs before and after adsorption over Be_12_O_12_. By the end, recovery time (*τ*) values were calculated to simulate the NBs desorption from the Be_12_O_12_. The current study intends to propose a promising nanocarrier for the NBs delivery process.

## Computational methods

In the realm of DFT calculations, the adsorption characteristics of NBs (i.e., NB-C, NB-G, NB-A, NB-T, and NB-U) over Be_12_O_12_ were investigated using the DFT/M06-2X method with 6–311+G** basis set, where no additional dispersion correction was applied [46–48]. By means of the Gaussian 09 package, all DFT calculations were performed [49]. For systems under study, geometrical optimization was executed. Cartesian atomic coordinates for the optimized structures are given in S1 Table. Frequency calculations were performed to confirm the optimized geometries are true minima. Electrostatic potential (ESP) analysis was investigated concerning the optimized NBs and Be_12_O_12_ systems. Therefore, molecular electrostatic potential (MEP) maps were generated and plotted at 0.002 au electron density envelopes [50]. Further, electrostatic potential extrema (*V*_s,min_/*V*_s,max_) values were calculated at 0.003 au isovalue using the Multiwfn 3.7 software [51]. The route sections of the employed DFT calculations are given in S2 Table.

**Fig 1:**
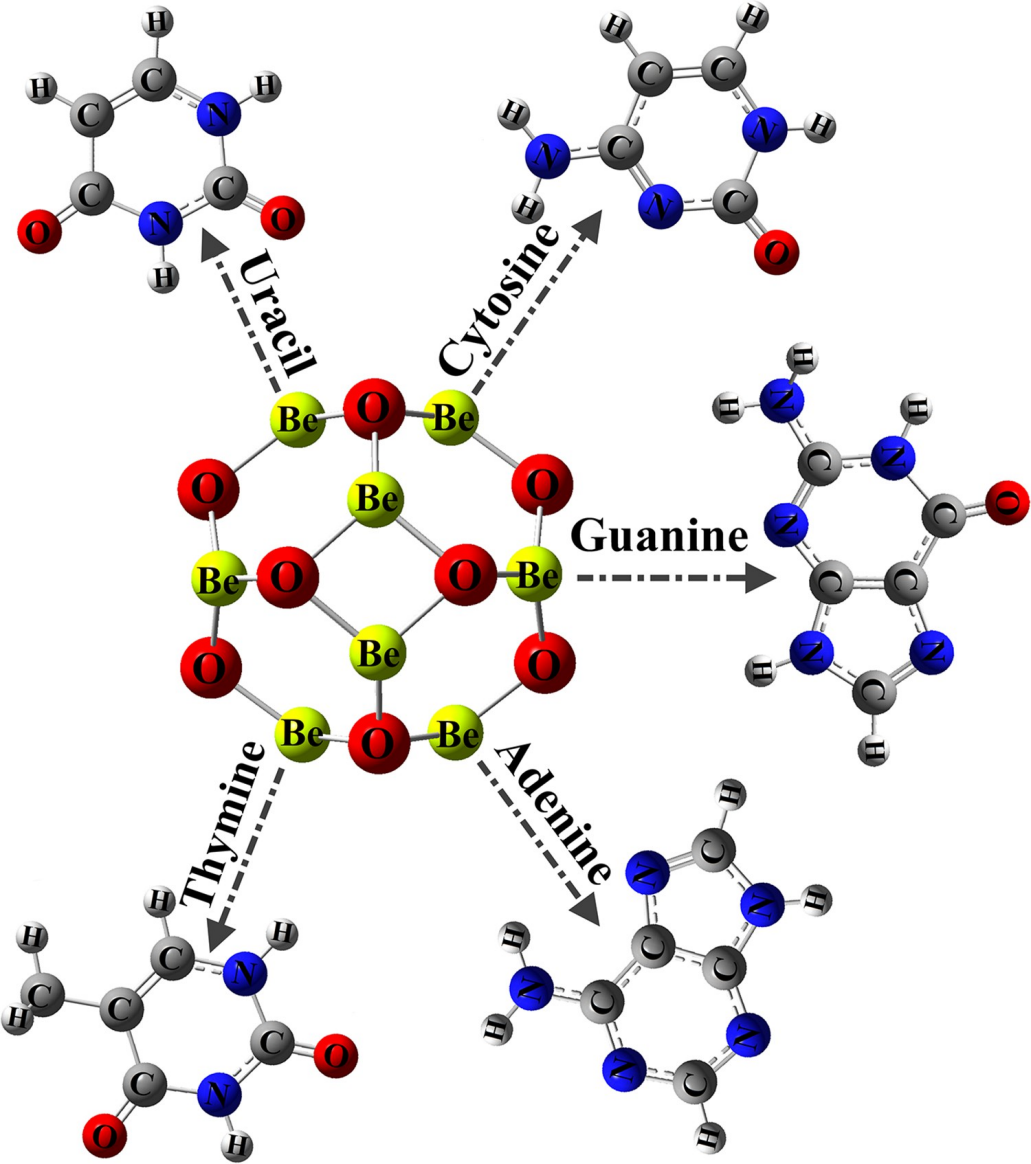
An illustration showing the adsorption of various NBs (Cytosine, guanine, adenine, thymine, and uracil) on Be_12_O_12_ nanocarrier.

Toward investigating the adsorption process, the NBs systems were oriented over Be_12_O_12_ through various adsorption sites (see Fig 1). Geometrical optimization was performed on NBs∙∙∙Be_12_O_12_ complexes within all possible configurations. The calculations employed SCF convergence criterion of 10^−8^ Hartrees, ultrafine integration grid, and the default optimization convergence criteria. The adsorption process was demonstrated by calculating the adsorption (*E*_ads_) and interaction (*E*_int_) energies. For further energy insights, interaction energies were estimated using the wB97X-D2/def2-TZVPD level of theory [52–54]. In energy calculations, the counterpoise corrected (CC) scheme proposed by Boys and Bernardi was utilized to reduce the basis set superposition error (BSSE) as follows [55]:

$$E_{ads}=E_{NBs{\cdots Be}_{12}O_{12}}-(E_{NBs}+E_{{Be}_{12}O_{12}})+E_{BSSE}$$ (1)

$$E_{int}=E_{NBs{\cdots Be}_{12}O_{12}}-(E_{NBs\;in\;complex}+E_{{Be}_{12}\;O_{12}\;in\;complex})+E_{BSSE}$$ (2)

where the energies for complex, isolated NBs, and isolated Be_12_O_12_ were represented by $E_{NBs∙∙∙{Be}_{12}O_{12}},E_{NBs}$, and $E_{{Be}_{12}O_{12}}$, respectively. Further, the energies for NBs and Be_12_O_12_, with geometries taken out from the optimized complexes, were identified as *E*_NBs in complex_ and $E_{{Be}_{12}O_{12}\;in\;complex}$, respectively.

For further energy insights, symmetry-adapted perturbation theory (SAPT) was implemented for all studied complexes. Accordingly, total energy was decomposed into its four physical forces that govern the adsorption process. Using PSI4 package [56], SAPT analysis was performed at the SAPT0 level [57]. Hence, the total energy (*E*^SAPT0^), electrostatic (*E*_elst_), exchange (*E*_exch_), induction (*E*_ind_), and dispersion (*E*_disp_) were computed according to the following equations [57]:

$$E^{SAPT0}=E_{elst}+E_{exch}+E_{ind}+E_{disp}$$ (3)

where

$$E_{elst}=E_{elst}^{\left(10\right)}$$ (4)

$$E_{exch}=E_{exch}^{\left(10\right)}$$ (5)

$$E_{ind}=E_{ind,resp}^{\left(20\right)}+E_{exch-ind,resp}^{\left(20\right)}+{\delta E}_{HF,resp}^{\left(2\right)}$$ (6)

$$E_{disp}=E_{disp}^{\left(20\right)}+E_{exch-disp}^{\left(20\right)}$$ (7)

The topology parameters and nature of the studied interactions were elucidated via the quantum theory of atoms in molecules (QTAIM) and the noncovalent interaction (NCI) index. Accordingly, the topological features were calculated along with bond paths (BPs) and bond critical points (BCPs) generation. Thus, the total energy density (H_b_), Laplacian (∇^2^*ρ*_b_), electron density (*ρ*_b_), kinetic electron density (G_b_), and the negative ratio of potential, kinetic electron energy density (−G_b_/V_b_), and local potential electron energy density (V_b_) were calculated. In conformity with NCI, the scatter 3D plots were mapped with colors ranging from blue (–0.035 au) to red (0.020 au) according to (λ^2^)*ρ* values. To perform the QTAIM and NCI analyses, the Multiwfn 3.7 software [51] was utilized, while the Visual Molecular Dynamics (VMD) program [58] was applied for visualization.

In order to understand the electronic features, the frontier molecular orbitals (FMOs) theory was investigated. In this regard, electronic patterns and energetic values of the highest occupied (HOMO) and the lowest unoccupied molecular orbitals (LUMO) were examined. According to the literature, the full range-separated functionals give better HOMO/LUMO energies [59, 60]. Meanwhile, the HOMO/LUMO calculations in the current study were performed at the same level of theory of optimization. From *E*_HOMO_ and *E*_LUMO_ values, the energy gap (*E*_gap_) and Fermi level (*E*_FL_) energies were calculated as follows:

$$E_{FL}=E_{HOMO}+\frac{E_{LUMO}-E_{HOMO}}{2}$$ (8)

$$E_{gap}=E_{LUMO}-E_{HOMO}$$ (9)

Toward an enhanced electronic investigation, further parameters such as ionization potential (*IP*), global softness (*S*), electron affinity (*EA*), global hardness (*η*), chemical potential (*μ*), electrophilicity index (*ω*), and work function (*Φ*) were calculated for the studied systems as follows:

$$IP\approx -E_{HOMO}$$ (10)

$$EA\approx -E_{LUMO}$$ (11)

$$\mu =\frac{E_{LUMO}+E_{HOMO}}{2}$$ (12)

$$\eta =\frac{E_{LUMO}-E_{HOMO}}{2}$$ (13)

$$S=\frac{1}{\eta }$$ (14)

$$\omega =\frac{\mu^{2}}{2\eta }$$ (15)

$$\Phi =V_{eL(+\infty )}-E_{FL}$$ (16)

Within Eq 16, the V_el_(+∞) identifies the vacuum level electrostatic potential (with a value near zero). Based on the energy gap (*E*_gap_), the electrical conductivity (σ) was examined as the following equation [61, 62]:

$$\sigma \;\alpha \;Exp\left(\frac{-E_{gap}}{kT}\right)$$ (17)

From the above-mentioned equation, k identifies the Boltzmann’s constant, and T represents the temperature. Additionally, further electronic insights were gained from the density of states (DOS) analysis. Therefore, DOS plots were generated for the considered systems using the GaussSum software [63].

In the framework of frequency calculations, thermodynamic parameters were evaluated for the studied NBs∙∙∙Be_12_O_12_ complexes. Accordingly, the Gibbs free energy (Δ*G*), enthalpy (Δ*H*), and entropy (Δ*S*) changes could be calculated as follows:

$$\Delta M=M_{NBs{\cdots Be}_{12}O_{12}}-(M_{NBs}+M_{{Be}_{12}O_{12}})+E_{BSSE}$$ (18)

ΔS=−(ΔG−ΔH)/T (19)

As described above, the Δ*M* is used to describe Δ*H* and Δ*G* parameters. Additionally, $M_{NBs{\cdots Be}_{12}O_{12}},M_{{Be}_{12}O_{12}}$, and *M*_NBs_ are used to identify the thermal parameters (i.e., *H* and *G*) for complexes, isolated Be_12_O_12_, and isolated NBs systems, respectively. Moreover, IR and Raman analyses were performed on the studied NBs before and after the adsorption. By the end, recovery time (*τ*) was elucidated to represent the desorption process and calculated as follows:

$$\tau =v^{-1}exp(-∆G/kT)$$ (20)

where the attempt frequency is represented by *v*^−1^ with a value of 10^−18^ s^–1^ [64].

## Results and discussion

### Electrostatic potential (ESP) analysis

**Fig 2:**
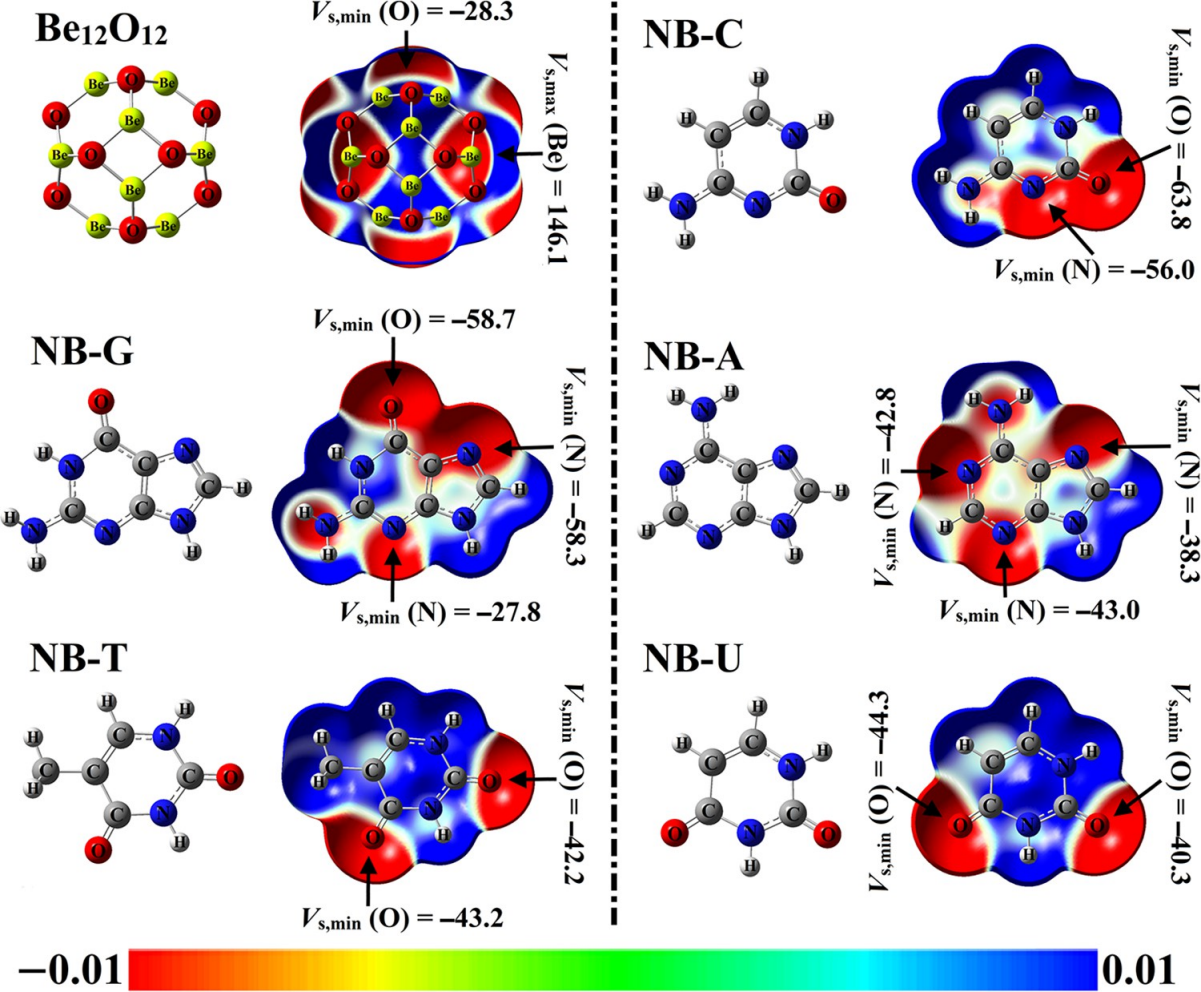
Optimized structures for all investigated NBs and Be_12_O_12_ systems, pairing with MEP maps and *V*_s,min_/*V*_s,max_ values (kcal/mol). MEP maps with color scale from red (–0.01 au) to blue (0.01 au).

ESP surface is an effective method to illustrate the positive and negative potentials over the molecular surface. In this regard, 3D maps of MEP were graphed along with calculating the *V*_s,max_ and *V*_s,min_ values. The optimized structures, MEP maps, and *V*_s,max_ / *V*_s,min_ values for all investigated NBs and Be_12_O_12_ systems were accumulated in Fig 2.

As delineated in Fig 2, in the case of NBs, various red regions were observed above O and N atoms with different sizes. For numerical evidence, the O and N atoms were found with *V*_s,min_ values up to –63.8 and –58.3 kcal/mol for NB-C and NB-G, respectively. Among the studied NBs, NB-C had the highest *V*_s,min_ values (i.e., –63.8 kcal/mol for O atom) compared to other analogs. For Be_12_O_12_, the blue color was observed over the Be atoms, indicating their attraction potentiality toward electronegative sites. Evidently, the Be atoms were characterized by *V*_s,max_ values of 146.1 kcal/mol.

### Adsorption process

**Fig 3:**
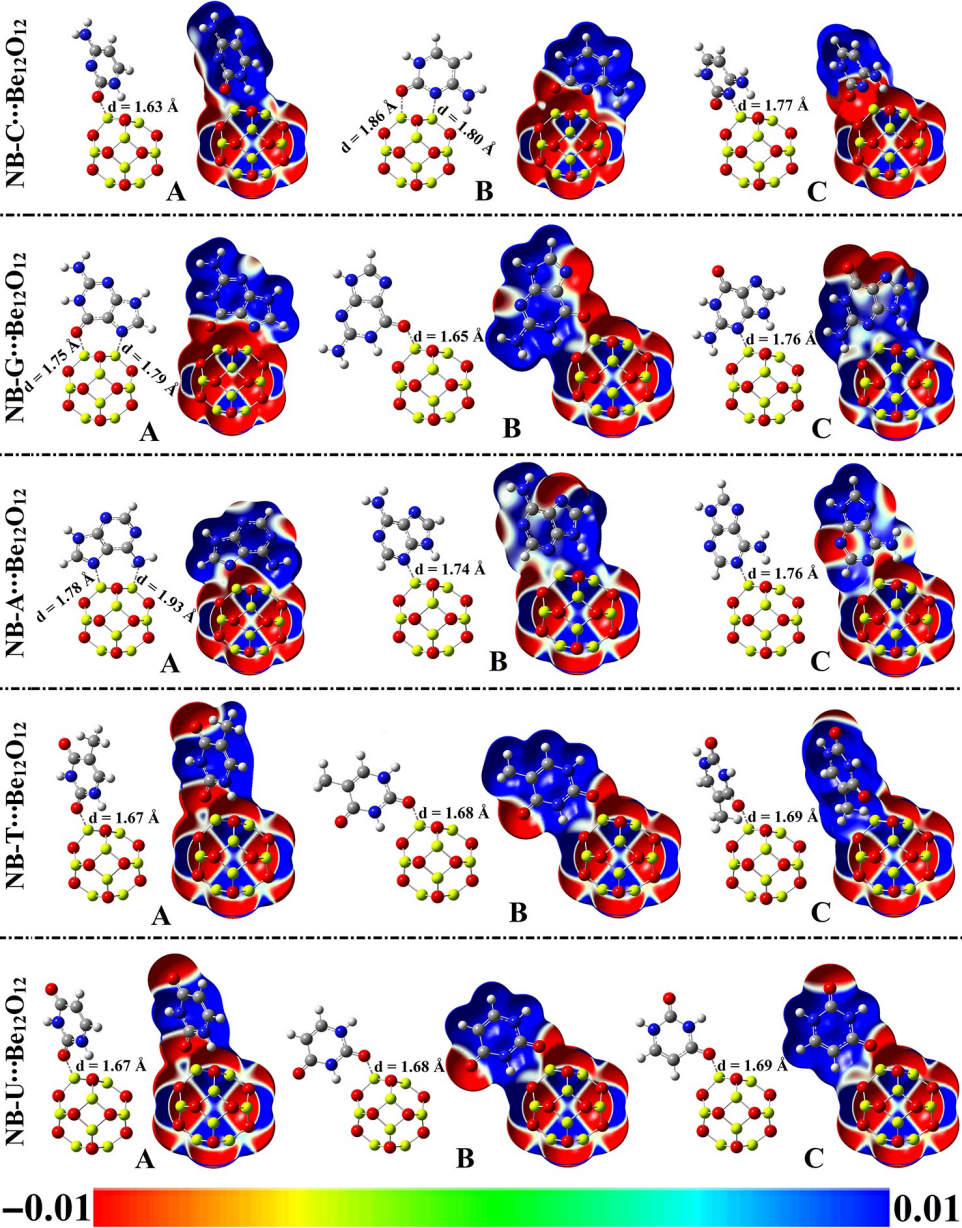
Optimized structures and MEP maps of NBs∙∙∙Be_12_O_12_ complexes within configurations A↔C.

In the scope of the adsorption process, NBs were oriented over Be_12_O_12_ through different adsorption sites, leading to various configurations (see Fig 3 and S1 Fig). Geometrical optimization was then performed for NBs∙∙∙Be_12_O_12_ complexes within configurations A↔C, followed by interaction (*E*_int_) and adsorption (*E*_ads_) energy calculations. Frequency calculations affirmed that all optimized complexes are true minima where no imaginary frequencies were found. Fig 3 contains optimized structures of NBs∙∙∙Be_12_O_12_ complexes within configurations A↔C, along with MEP maps. Table 1 summarizes the calculated *E*_int_ and *E*_ads_ energies, as well as equilibrium distances (d).

**Table 1:** Obtained values of intermolecular distances (d, Å) along with interaction (*E*_int_, kcal/mol) and adsorption (*E*_ads_, kcal/mol) energies for NBs∙∙∙Be_12_O_12_ complexes within configurations A↔C.

Complex	Configuration	Bond	d	*E*_int_	*E*_int_*^a^	*E*_ads_
**NB-C∙∙∙Be_12_O_12_**	A	O∙∙∙Be	1.63	–53.04	–49.98	–35.32
	B	O∙∙∙Be	1.86	–51.20	–45.69	–33.59
		N∙∙∙Be	1.80			
	C	N∙∙∙Be	1.77	–46.07	–43.17	–33.14
**NB-G∙∙∙Be_12_O_12_**	A	O∙∙∙Be	1.75	–51.45	–46.28	–38.30
		N∙∙∙Be	1.79			
	B	O∙∙∙Be	1.65	–47.38	–44.97	–32.28
	C	N∙∙∙Be	1.76	–46.72	–44.53	–34.40
**NB-A∙∙∙Be_12_O_12_**	A	N∙∙∙Be	1.78	–46.01	–41.27	–29.83
		N∙∙∙Be	1.93			
	B	N∙∙∙Be	1.74	–45.00	–42.73	–33.66
	C	N∙∙∙Be	1.76	–43.93	–41.43	–32.88
**NB-T∙∙∙Be_12_O_12_**	A	O∙∙∙Be	1.67	–44.37	–41.50	–30.09
	B	O∙∙∙Be	1.68	–39.17	–36.25	–27.74
	C	O∙∙∙Be	1.69	–32.01	–29.45	–22.16
**NB-U∙∙∙Be_12_O_12_**	A	O∙∙∙Be	1.67	–43.83	–41.03	–29.62
	B	O∙∙∙Be	1.68	–38.18	–35.29	–27.05
	C	O∙∙∙Be	1.69	–33.35	–30.44	–23.63

^*a*^ Interaction energies (*E*_int_*) calculated at wB97X-D2/def2-TZVPD level of theory.

From the data in Table 1, the favorable performance of Be_12_O_12_ toward adsorbing NBs was assured by negative *E*_int_ and *E*_ads_ values. Concurrent with Fig 3 and Table 1, short intermolecular distances (d) also confirmed the occurrence of the adsorption process within NBs∙∙∙Be_12_O_12_ complexes. Based on numerical values, intermolecular distances (d) varied from 1.63 to 1.93 Å for NB-C∙∙∙ and NB-A∙∙∙Be_12_O_12_ complexes within configuration A, respectively. Out of all studied complexes, NB-C∙∙∙Be_12_O_12_ complexes showed the most negative *E*_int_ value and the shortest intermolecular distance (d) compared to other analogs. For instance, *E*_int_ was found with values of –53.04, –51.45, –46.01, –44.37, and –43.83 kcal/mol for NB-C∙∙∙, NB-G∙∙∙, NB-A∙∙∙, NB-T∙∙∙, and NB-U∙∙∙Be_12_O_12_ complexes within configuration A, respectively. Generally, *E*_int_ and *E*_ads_ values were inversely correlated with d values, where *E*_int_ and *E*_ads_ values increased as d values decreased. For instance, *E*_int_ values were –44.37, –39.17, and –32.01 kcal/mol, while d values were 1.67, 1.68, and 1.69 Å for NB-T∙∙∙Be_12_O_12_ complexes within configurations A, B, and C, respectively. In most cases, configuration A had more preferable energy values than other configurations, where the *E*_int_ values increased in the following order: C < B < A. Numerically, *E*_int_ energies were found to be –46.01, –45.00, and –43.93 kcal/mol for NB-A∙∙∙Be_12_O_12_ complexes within configurations A, B, and C, respectively. Accordingly, the significant energy values found for configuration A compared to other analogs could be attributed to its short intermolecular distances along with the role of secondary interactions, as shown in Fig 3. Further, interaction energies (*E*_int_*) were calculated at the wB97X-D2/def2-TZVPD level of theory (Table 1). Notably, the correlation coefficient (*R*^2^) value between *E*_int_ and *E*_int_* was 0.97, demonstrating a significant resemblance between the calculated values using the M06-2X/6-311+G** and wB97X-D2/def2-TZVPD levels of theory. Accordingly, the calculated *E*_int_* values followed the same trend as *E*_int_ values where the energy enhanced in the following trend NB-U∙∙∙ < NB-T∙∙∙ < NB-A∙∙∙ < NB-G∙∙∙ < NB-C∙∙∙Be_12_O_12_ complexes. To sum up, the obtained energies ensured the preferability of the NBs adsorption process over Be_12_O_12_.

### SAPT calculations

An energy decomposition analysis was implemented for the NBs∙∙∙Be_12_O_12_ complexes using symmetry-adapted perturbation theory (SAPT). By performing SAPT analysis, the total energy (*E*^SAPT0^) and its main physical components were calculated for the considered complexes (Table 2).

**Table 2:** Summarized values of total energy (*E*^SAPT0^), electrostatic (*E*_elst_), induction (*E*_ind_), dispersion (*E*_disp_), and exchange (*E*_exch_) energies for NBs∙∙∙Be_12_O_12_ complexes within configurations A↔C. All values are in kcal/mol.

Complex	Configuration	*E* ^SAPT0^	*E*_elst_	*E*_ind_	*E*_disp_	*E*_exch_
**NB-C∙∙∙Be_12_O_12_**	A	–56.67	–77.17	–37.30	–14.05	71.85
	B	–52.31	–99.88	–34.65	–21.76	103.98
	C	–47.63	–70.21	–31.14	–16.54	70.26
**NB-G∙∙∙Be_12_O_12_**	A	–54.54	–88.92	–35.72	–20.52	90.62
	B	–51.26	–70.98	–34.95	–14.04	68.71
	C	–48.77	–75.97	–30.15	–17.66	75.02
**NB-A∙∙∙Be_12_O_12_**	A	–47.69	–87.37	–33.28	–21.71	94.66
	B	–46.56	–69.34	–27.65	–14.94	65.37
	C	–45.30	–69.98	–27.95	–15.53	68.16
**NB-T∙∙∙Be_12_O_12_**	A	–47.37	–67.07	–32.55	–13.50	65.74
	B	–41.90	–60.46	–28.40	–12.72	59.68
	C	–34.07	–42.91	–23.09	–12.80	44.72
**NB-U∙∙∙Be_12_O_12_**	A	–46.94	–66.97	–32.72	–13.53	66.28
	B	–40.92	–59.62	–28.07	–12.69	59.46
	C	–35.90	–47.63	–23.78	–11.03	46.54

As found in Table 2, negative values of total energy (*E*^SAPT0^) indicated the promising loading process of NBs over the Be_12_O_12_ nanocarrier. It turns out from Table 2 that electrostatic (*E*_elst_), induction (*E*_ind_), and dispersion (*E*_disp_) forces promoted the adsorption process within all studied complexes. Further, electrostatic (*E*_elst_) mainly dominated the studied adsorption process with *E*_elst_ values up to –99.88 kcal/mol for NB-C∙∙∙Be_12_O_12_ complex within configuration B. Nevertheless, the unfavorable role of exchange (*E*_exch_) forces in the studied adsorption process was observed. For example, *E*_elst_, *E*_ind_, *E*_disp_, and *E*_exch_ forces for NB-C∙∙∙Be_12_O_12_ complex within configuration A were found with values of –77.17, –37.30, –14.05, and 71.85 kcal/mol, respectively. In conformity with data in Table 1, *E*^SAPT0^ energies also followed a similar trend to *E*_int_ values, and the overall energies decreased in the order of NB-C∙∙∙ > NB-G∙∙∙ > NB-A∙∙∙ > NB-T∙∙∙ > NB-U∙∙∙Be_12_O_12_. For instance, *E*_int_ (*E*^SAPT0^) energies were found with values of –53.04 (–56.67), –51.45 (–54.54), –46.01 (–47.69), –44.37 (–47.37), and –43.83 (–46.94) kcal/mol for NB-C∙∙∙, NB-G∙∙∙, NB-A∙∙∙, NB-T∙∙∙, and NB-U∙∙∙Be_12_O_12_ complexes within configuration A, respectively.

### QTAIM and NCI calculations

**Fig 4:**
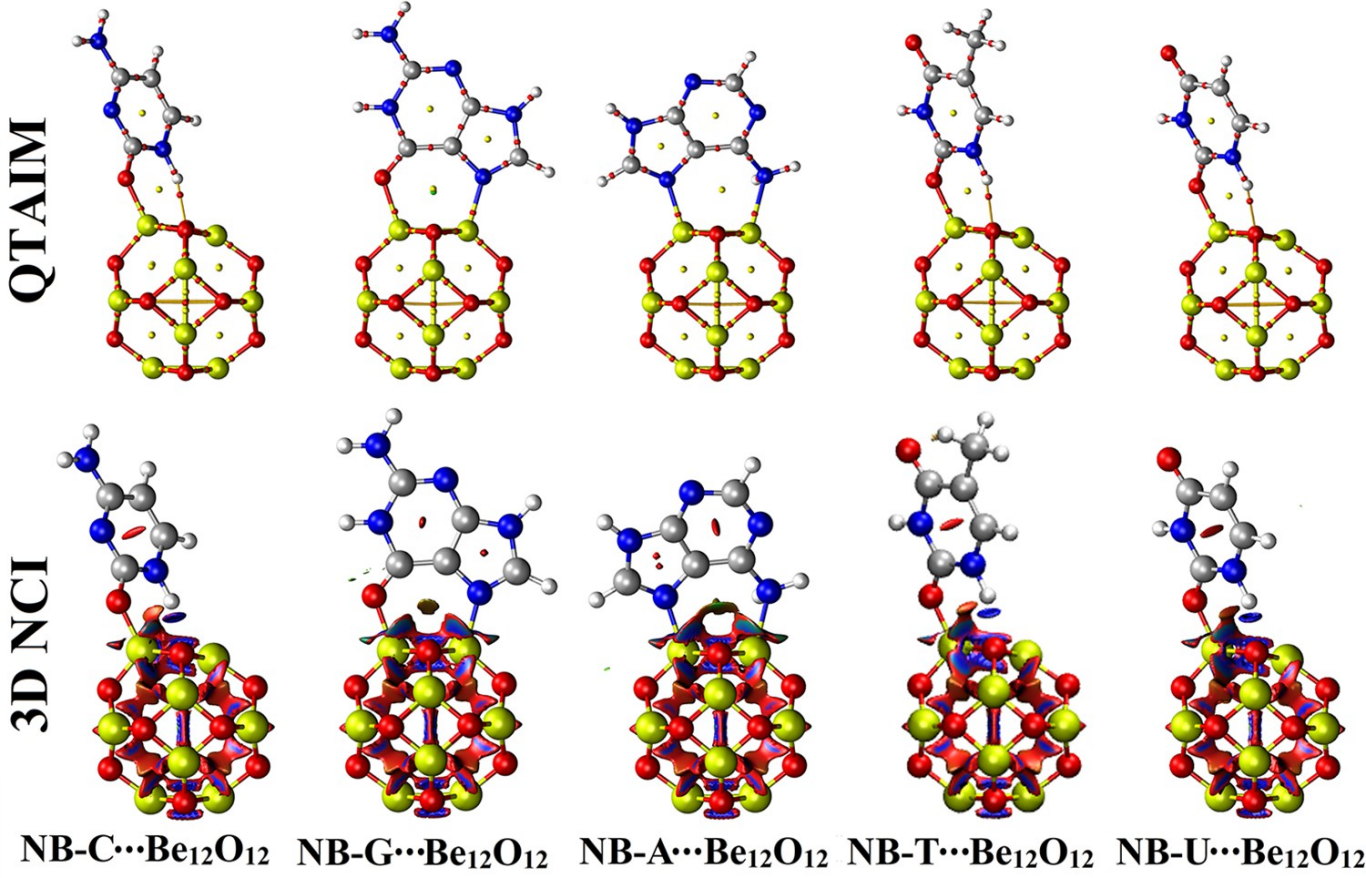
3D graphs of QTAIM and NCI for the optimized NBs∙∙∙Be_12_O_12_ complexes within configuration A.

QTAIM and NCI index analyses are proposed as illustrative tools for the occurrence of the adsorption process. Accordingly, the QTAIM and NCI analyses were performed for the studied NBs∙∙∙Be_12_O_12_ complexes within configurations A↔C. Fig 4 collects the generated plots for NBs∙∙∙Be_12_O_12_ complexes within configuration A; meanwhile, S2 Fig gathers the plots for configurations B and C. Further, the 2D NCI graphs were extracted for all studied complexes and collected in S3 Fig. The topological features were also calculated and gathered in Table 3.

**Table 3:** Calculated topological parameters (in au) for the optimized NBs∙∙∙Be_12_O_12_ complexes within configurations A↔C.

Complex	Configuration	Bond	∇^2^*ρ*_b_	*ρ*_b_	V_b_	H_b_	G_b_	−G_b_/V_b_
**NB-C∙∙∙Be**_**12**_ **O**_**12**_	**A**	O∙∙∙Be	0.5068	0.0683	−0.1191	0.0038	0.1229	1.0319
		H∙∙∙O	0.1534	0.0496	−0.0509	−0.0063	0.0446	0.8770
	**B**	O∙∙∙Be	0.2317	0.0375	−0.0558	0.0011	0.0569	1.0191
		N∙∙∙Be	0.3229	0.0531	−0.0838	−0.0016	0.0823	0.9814
		H∙∙∙O	0.0861	0.0232	−0.0178	0.0018	0.0197	1.1036
	**C**	N∙∙∙Be	0.3388	0.0595	−0.0923	−0.0038	0.0885	0.9589
		H∙∙∙O	0.1417	0.0400	−0.0386	−0.0016	0.0370	0.9591
**NB-G∙∙∙Be**_**12**_ **O**_**12**_	**A**	O∙∙∙Be	0.3502	0.0475	−0.0764	0.0056	0.0820	1.0727
		N∙∙∙Be	0.3329	0.0527	−0.0832	0.0000	0.0832	1.0001
	**B**	O∙∙∙Be	0.4762	0.0644	−0.1104	0.0043	0.1147	1.0394
		H∙∙∙O	0.1505	0.0473	−0.0477	−0.0051	0.0427	0.8940
	**C**	N∙∙∙Be	0.3475	0.0609	−0.0954	−0.0043	0.0911	0.9554
		H∙∙∙O	0.1149	0.0297	−0.0258	0.0015	0.0273	1.0562
		H∙∙∙O	0.1079	0.0272	−0.0229	0.0020	0.0249	1.0892
**NB-A∙∙∙Be**_**12**_ **O**_**12**_	**A**	N∙∙∙Be	0.3311	0.0537	−0.0844	−0.0008	0.0836	0.9906
		N∙∙∙Be	0.2137	0.0374	−0.0532	0.0001	0.0533	1.0019
	**B**	N∙∙∙Be	0.3645	0.0633	−0.1005	−0.0047	0.0958	0.9532
		H∙∙∙O	0.1066	0.0272	−0.0227	0.0020	0.0247	1.0865
	**C**	N∙∙∙Be	0.3481	0.0616	−0.0964	−0.0047	0.0917	0.9514
		H∙∙∙O	0.1245	0.0322	−0.0290	0.0011	0.0301	1.0366
**NB-T∙∙∙Be**_**12**_ **O**_**12**_	**A**	O∙∙∙Be	0.4574	0.0615	−0.1043	0.0050	0.1093	1.0483
		H∙∙∙O	0.1491	0.0461	−0.0462	−0.0044	0.0417	0.9037
	**B**	O∙∙∙Be	0.4419	0.0589	−0.0991	0.0057	0.1048	1.0573
		H∙∙∙O	0.1331	0.0375	−0.0351	−0.0009	0.0342	0.9743
	**C**	O∙∙∙Be	0.4223	0.0540	−0.0905	0.0076	0.0980	1.0835
		H∙∙∙O	0.0371	0.0110	−0.0069	0.0012	0.0081	1.1751
		H∙∙∙O	0.0366	0.0109	−0.0068	0.0012	0.0080	1.1738
**NB-U∙∙∙Be**_**12**_ **O**_**12**_	**A**	O∙∙∙Be	0.4515	0.0607	−0.1025	0.0052	0.1077	1.0506
		H∙∙∙O	0.1513	0.0482	−0.0489	−0.0055	0.0434	0.8867
	**B**	O∙∙∙Be	0.4336	0.0578	−0.0969	0.0058	0.1026	1.0596
		H∙∙∙O	0.1349	0.0383	−0.0361	−0.0012	0.0349	0.9671
	**C**	O∙∙∙Be	0.4273	0.0561	−0.0937	0.0066	0.1003	1.0700
		H∙∙∙O	0.0656	0.0175	−0.0121	0.0021	0.0143	1.1762

As apparently shown in Fig 4 and S2 Fig, the adsorption process within NBs∙∙∙Be_12_O_12_ complexes was verified through the BPs and BCPs formed between the interacted molecules. This finding provided a compelling depiction of the potentiality of Be_12_O_12_ to adsorb NBs via different interactions (i.e., N∙∙∙ and O∙∙∙Be interactions). Notoriously, BPs and BCPs were also observed between the H atom of NBs and O atoms of Be_12_O_12_, highlighting the essential role of these interactions in stabilizing the studied adsorption process. Turning to 2D NCI spikes (S3 Fig), the sign (λ_2_)*ρ* values were found to be less than 0.01 au in all studied complexes, confirming the attractive interactions among NBs and Be_12_O_12_.

As previously reported [65], the H_b_ and ∇^2^*ρ*_b_ functions could be used to classify the strength of intermolecular interactions. Therefore, the interactions are characterized as strong covalent, weak electrostatic, and partial covalent and electrostatic for {H_b_ < 0, ∇^2^*ρ*_b_ < 0}, {H_b_ > 0, ∇^2^*ρ*_b_ > 0}, and {H_b_ < 0, ∇^2^*ρ*_b_ > 0}, respectively.

Table 3 indicates the interactions within NBs∙∙∙Be_12_O_12_ complexes to be electrostatic and partially covalent according to ∇^2^*ρ*_b_, *ρ*_b_, H_b_, and −G_b_/V_b_ values. For NB-A∙∙∙Be_12_O_12_ complex within configuration C, the obtained values of ∇^2^*ρ*_b_, *ρ*_b_, H_b_, and −G_b_/V_b_ were found to be 0.3481, 0.0616, −0.0047, and 0.9514 au, respectively. Generally, the ∇^2^*ρ*_b_ and *ρ*_b_ values were found to be in the same line with the energetic patterns (Table 1).

### Electronic parameters

**Fig 5:**
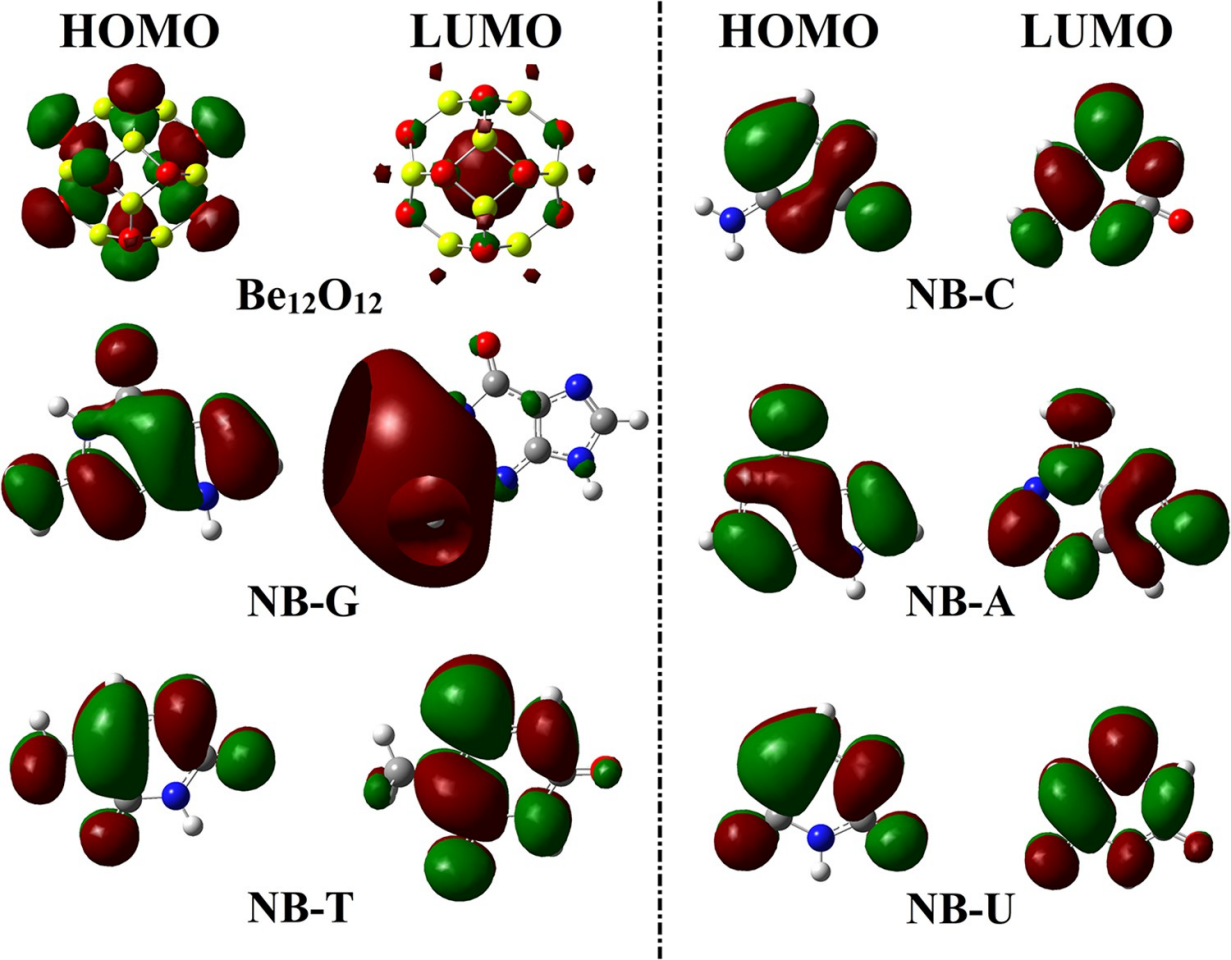
Visualization of HOMO and LUMO isosurfaces around the optimized NBs and Be_12_O_12_ molecular surfaces.

**Fig 6:**
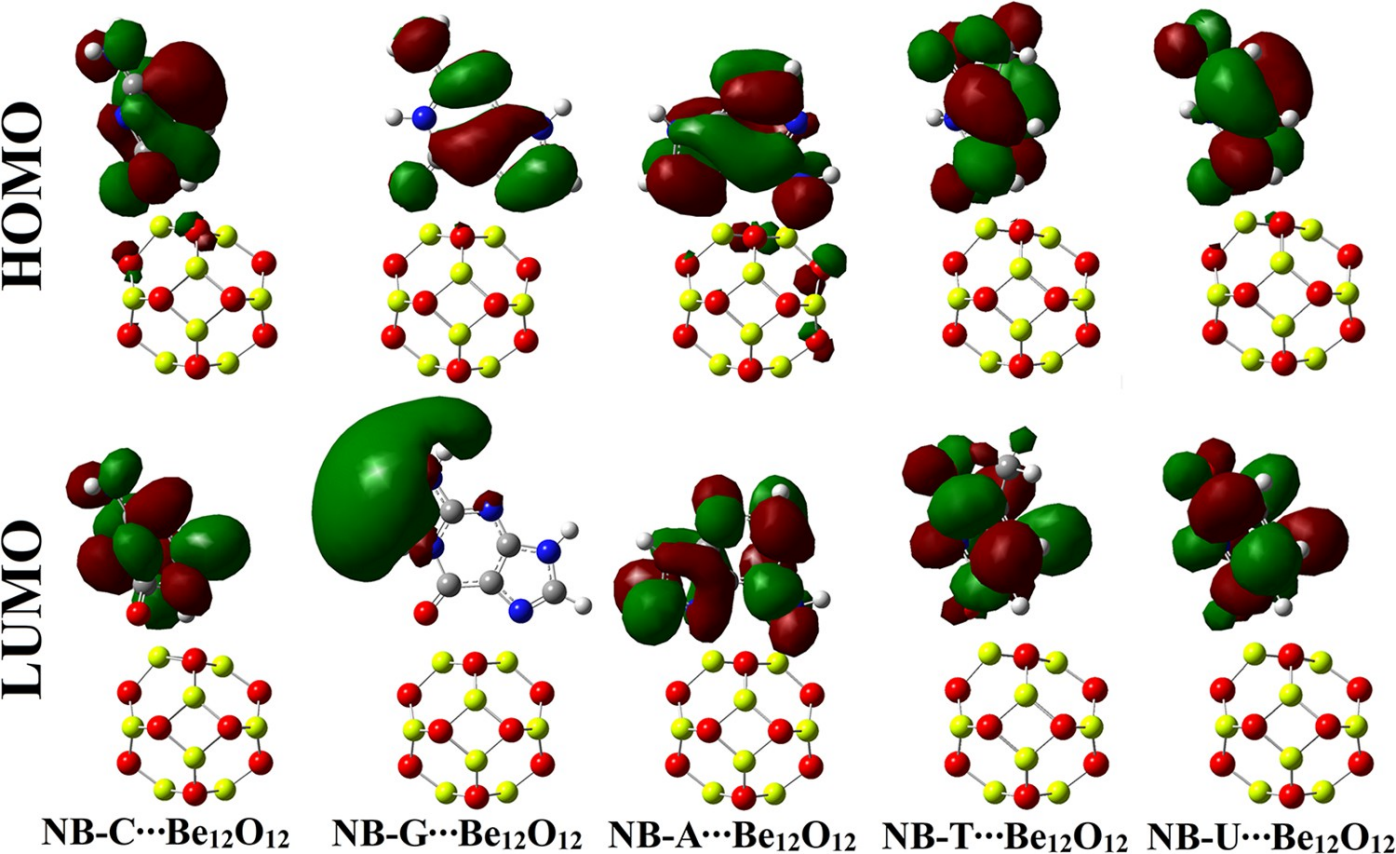
The HOMO and LUMO plots over the optimized NBs∙∙∙Be_12_O_12_ complexes within configuration A.

Toward electronic illustration, FMOs theory was conducted for NBs∙∙∙Be_12_O_12_ complexes under investigation. In this respect, the HOMO and LUMO levels were generated for the isolated systems and collected in Fig 5. Further, Fig 6 shows the HOMO and LUMO orbitals for the NBs∙∙∙Be_12_O_12_ complexes within configuration A, whereas S4 Fig shows the corresponding plots for configurations B and C. The energies of molecular orbitals (i.e., *E*_HOMO_ and *E*_LUMO_) were calculated along with their related parameters (i.e., *E*_gap_ and *E*_FL_) and tabulated in Table 4.

**Table 4:** The obtained *E*_HOMO_, *E*_LUMO_, *E*_gap_, and *E*_FL_ values (eV) for the NBs, Be_12_O_12_, and their complexes.

System	Configuration	*E*_HOMO_	*E*_LUMO_	*E*_gap_	*E*_FL_
**NB-C**		–8.04	–0.26	7.77	–4.15
**NB-G**		–7.38	–0.29	7.09	–3.84
**NB-A**		–7.62	0.05	7.67	–3.79
**NB-T**		–8.32	–0.46	7.86	–4.39
**NB-U**		–8.71	–0.58	8.13	–4.64
Be_12_O_12_		–10.60	–0.67	9.93	–5.63
**NB-C∙∙∙Be_12_O_12_**	A	–8.90	–0.86	8.04	–4.88
	B	–8.95	–1.37	7.58	–5.16
	C	–8.80	–1.01	7.79	–4.91
**NB-G∙∙∙Be_12_O_12_**	A	–8.50	–0.97	7.54	–4.74
	B	–7.97	–0.73	7.23	–4.35
	C	–7.81	–0.82	7.00	–4.31
**NB-A∙∙∙Be_12_O_12_**	A	–9.21	–1.32	7.89	–5.27
	B	–8.26	–0.69	7.57	–4.47
	C	–8.20	–0.50	7.70	–4.35
**NB-T∙∙∙Be_12_O_12_**	A	–8.88	–0.97	7.91	–4.92
	B	–8.99	–0.96	8.03	–4.98
	C	–9.04	–1.47	7.57	–5.25
**NB-U∙∙∙Be_12_O_12_**	A	–9.24	–1.10	8.14	–5.17
	B	–9.37	–1.10	8.27	–5.24
	C	–9.44	–1.53	7.90	–5.48

According to Fig 5, the HOMO and LUMO levels mainly covered O and Be atoms of Be_12_O_12_, respectively. For NBs, the HOMO and LUMO patterns were localized over nucleophilic (i.e., O and N) and electrophilic (i.e., H and C) sites, respectively. Turning to NBs∙∙∙Be_12_O_12_ complexes, a remarkable redistribution in HOMO and LUMO levels was observed, demonstrating the occurrence of the adsorption process (see Fig 6 and S4 Fig). Further, the observed changes after NBs adsorption highlighted the effect of Be_12_O_12_ on the electronic distribution of NBs. It was essential to accentuate that the observed changes in HOMO and LUMO distributions of NBs after the adsorption over Be_12_O_12_ strongly related to the occurrence of charge transfer within the NBs∙∙∙Be_12_O_12_ complexes.

From data in Table 4, *E*_HOMO_ and *E*_LUMO_ for NBs were found with different values, demonstrating the effect of molecular structure on electronic nature. For instance, NB-C, NB-G, NB-A, NB-T, and NB-U demonstrated *E*_HOMO_ values of –8.04, –7.38, –7.62, –8.32, and –8.71 eV, respectively. After the NBs adsorption over Be_12_O_12_, the electronic parameters for Be_12_O_12_ were changed, denoting the favorable NBs adsorption over Be_12_O_12_. For instance, *E*_HOMO_ values for Be_12_O_12_ were found to be –10.60 eV and changed to –8.90, –8.95, and –8.80 eV in NB-C∙∙∙Be_12_O_12_ complexes within configurations A, B, and C, respectively. In this respect, *E*_gap_ values of Be_12_O_12_ decreased due to the adsorption process of NBs, which in turn increased the conductivity (σ). Enhanced conductivity promoted the application of Be_12_O_12_ as electrochemical biosensors for NBs. Numerically, Be_12_O_12_ had *E*_gap_ = 9.93 eV that decreased to 8.04 eV for NB-C∙∙∙Be_12_O_12_ complex within configuration A.

### Global indices of reactivity

Based on the undisputed role of electronic parameters, global reactivity indices were calculated for the isolated NBs and Be_12_O_12_, along with their combined complexes. Table 5 presents the values of global reactivity indices for NBs and Be_12_O_12_, along with their complexes.

**Table 5:** Calculated parameters for the isolated NBs and Be_12_O_12_ systems and their corresponding NBs∙∙∙Be_12_O_12_ complexes within configurations A↔C.

System	Configuration	*IP* (eV)	*EA* (eV)	*μ* (eV)	*η* (eV)	*S* (eV^-1^)	*ω* (eV)	*Φ* (eV)
**NB-C**		8.04	0.26	–4.15	3.89	0.26	2.21	4.15
**NB-G**		7.38	0.29	–3.84	3.54	0.28	2.08	3.84
**NB-A**		7.62	–0.05	–3.79	3.83	0.26	1.87	3.79
**NB-T**		8.32	0.46	–4.39	3.93	0.25	2.46	4.39
**NB-U**		8.71	0.58	–4.64	4.06	0.25	2.65	4.64
Be_12_O_12_		10.60	0.67	–5.63	4.97	0.20	3.19	5.63
**NB-C∙∙∙Be_12_O_12_**	A	8.90	0.86	–4.88	4.02	0.25	2.97	4.88
	B	8.95	1.37	–5.16	3.79	0.26	3.51	5.16
	C	8.80	1.01	–4.91	3.90	0.26	3.09	4.91
**NB-G∙∙∙Be_12_O_12_**	A	8.50	0.97	–4.74	3.77	0.27	2.98	4.74
	B	7.97	0.73	–4.35	3.62	0.28	2.62	4.35
	C	7.81	0.82	–4.31	3.50	0.29	2.66	4.31
**NB-A∙∙∙Be_12_O_12_**	A	9.21	1.32	–5.27	3.94	0.25	3.52	5.27
	B	8.26	0.69	–4.47	3.78	0.26	2.65	4.47
	C	8.20	0.50	–4.35	3.85	0.26	2.45	4.35
**NB-T∙∙∙Be_12_O_12_**	A	8.88	0.97	–4.92	3.96	0.25	3.06	4.92
	B	8.99	0.96	–4.98	4.02	0.25	3.09	4.98
	C	9.04	1.47	–5.25	3.78	0.26	3.65	5.25
**NB-U∙∙∙Be_12_O_12_**	A	9.24	1.10	–5.17	4.07	0.25	3.28	5.17
	B	9.37	1.10	–5.24	4.13	0.24	3.32	5.24
	C	9.44	1.53	–5.48	3.95	0.25	3.81	5.48

From Table 5, significant variations in the calculated parameters for Be_12_O_12_ were observed following the adsorption of NBs. For instance, *IP* was found with a value of 10.60 eV for isolated Be_12_O_12_ and changed to 8.90, 8.95, and 8.80 eV for NB-C∙∙∙Be_12_O_12_ complexes within configurations A, B, and C, respectively. Further, *η* values of Be_12_O_12_ were decreased upon the adsorption process of NBs, which, in turn, enhanced the *S* values. Numerically, *η* values were found to be 4.97 and 4.02/3.79/3.90 eV for Be_12_O_12_ and NB-C∙∙∙Be_12_O_12_ complexes within A/B/C configurations, respectively.

### DOS analysis

**Fig 7:**
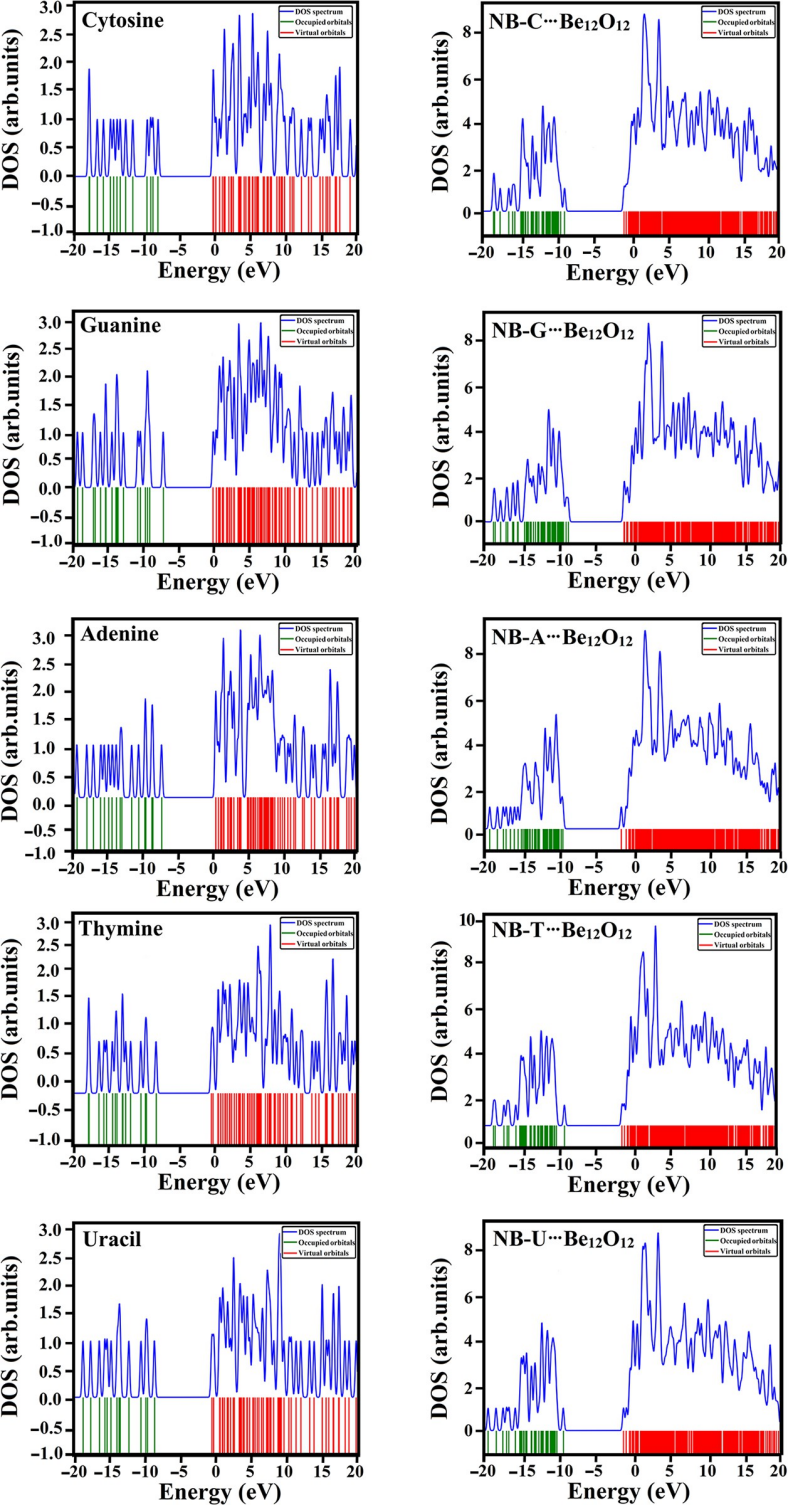
DOS plots for the isolated NBs and their related NBs∙∙∙Be_12_O_12_ complexes within configuration A.

For further electronic aspects, the density of state (DOS) analysis was performed for NBs before and after the adsorption process over Be_12_O_12_. Fig 7 gatherers the DOS plots for NBs and their related NBs∙∙∙Be_12_O_12_ complexes within configuration A. Further, S5 Fig gathered the DOS plots of NBs∙∙∙Be_12_O_12_ complexes within configurations C and D.

As shown in Fig 7 and S5 Fig, the DOS plots demonstrated the alterations in electronic features for NBs upon the adsorption over Be_12_O_12_. Obviously, the NBs adsorption over the Be_12_O_12_ led to a displacement of the HOMO and LUMO peaks. For instance, *E*_HOMO_ and *E*_LUMO_ values for NB-C were –8.04 and –0.26 eV, while for NB-C∙∙∙Be_12_O_12_ complex within configuration A were –8.90 and –0.86 eV, respectively. By the end, the DOS analysis affirmed the occurrence of the adsorption process within NBs∙∙∙Be_12_O_12_ complexes that are in line with the above-mentioned analyses.

### Thermodynamic parameters

In the quest for thermal comprehension, thermodynamic parameters were estimated for all NBs∙∙∙Be_12_O_12_ complexes and tabulated in Table 6.

**Table 6:** Thermodynamic parameters (in kcal/mol) for NBs∙∙∙Be_12_O_12_ complexes within configurations A↔C.

Complex	Configuration	Δ*G*	Δ*H*	Δ*S*
**NB-C∙∙∙Be_12_O_12_**	A	–21.95	–33.60	–0.039
	B	–17.78	–31.35	–0.046
	C	–18.67	–31.05	–0.042
**NB-G∙∙∙Be_12_O_12_**	A	–23.31	–37.47	–0.048
	B	–19.07	–31.32	–0.041
	C	–20.02	–32.98	–0.043
**NB-A∙∙∙Be_12_O_12_**	A	–13.46	–28.05	–0.049
	B	–19.36	–32.16	–0.043
	C	–17.97	–31.17	–0.044
**NB-T∙∙∙Be_12_O_12_**	A	–16.75	–29.10	–0.041
	B	–14.32	–26.58	–0.041
	C	–9.05	–20.72	–0.039
**NB-U∙∙∙Be_12_O_12_**	A	–16.18	–28.59	–0.042
	B	–13.67	–25.87	–0.041
	C	–10.50	–22.16	–0.039

According to Table 6, the adsorption process within NBs∙∙∙Be_12_O_12_ complexes was spontaneous and exothermic based on negative values of Δ*G* and Δ*H*, respectively. Further, Δ*S* was found with small negative values compared to Δ*G* and Δ*H*. Numerically, for NB-C∙∙∙Be_12_O_12_ complex within configuration A, Δ*G*, Δ*H*, and Δ*S* were found with values of –21.95, –33.60, and –0.039 kcal/mol, respectively. Further, the most negative Δ*G* value was found for configuration A, which is in line with the energy affirmations (Table 1). For example, Δ*G* values were –16.18, –13.67, and –10.50 kcal/mol for NB-U∙∙∙Be_12_O_12_ complexes within configurations A, B, and C, respectively. To sum up, the obtained parameters affirmed the spontaneous and exothermic natures of the investigated adsorption process.

### IR and Raman spectra

**Fig 8:**
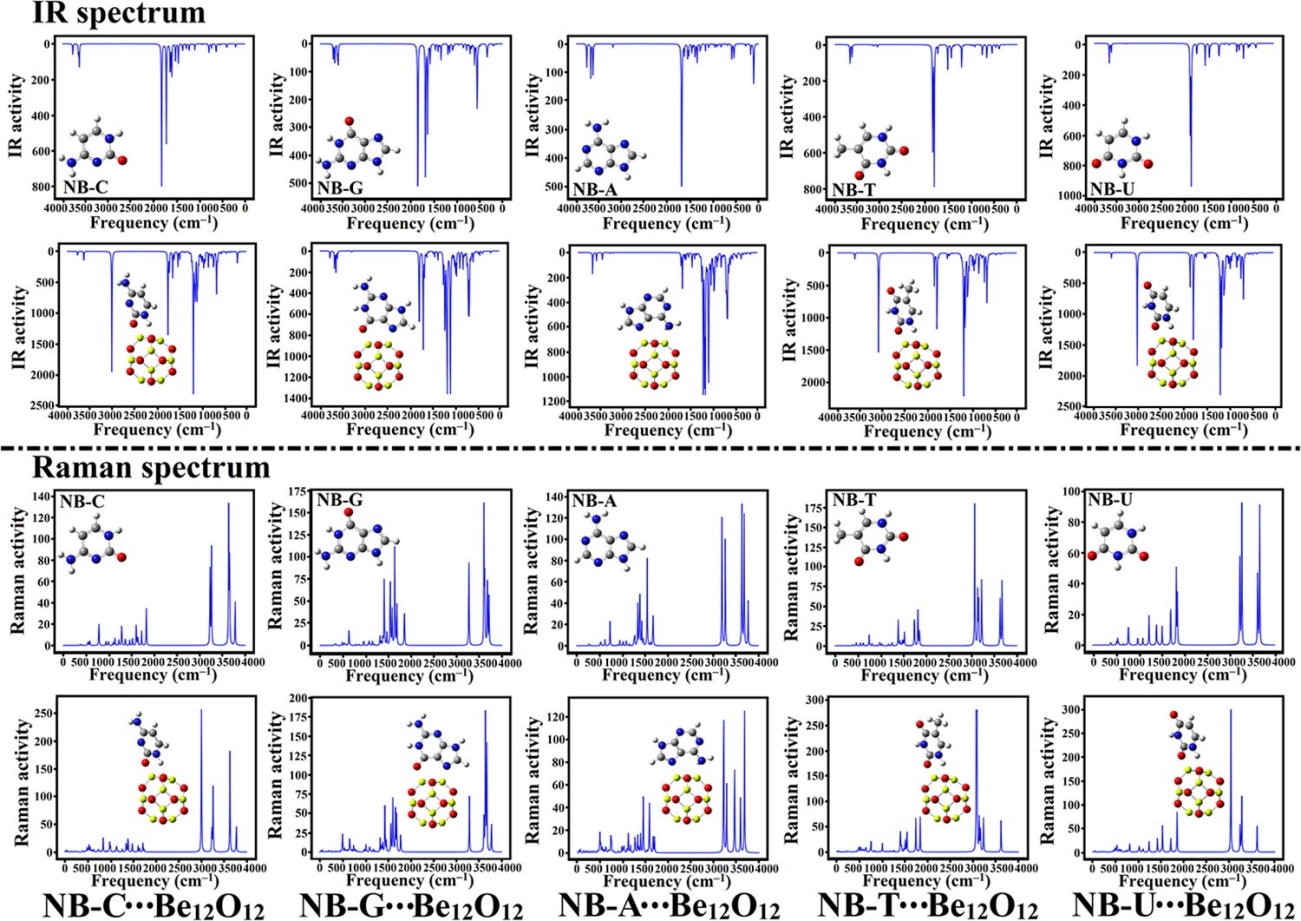
Plotted 2D IR and Raman spectra for the NBs and NBs∙∙∙Be_12_O_12_ complexes within configuration A.

Infrared (IR) and Raman spectra were visualized and plotted for isolated NBs and their related complexes with Be_12_O_12_. The obtained spectra for isolated NBs and NBs∙∙∙Be_12_O_12_ complexes within configuration A were extracted (Fig 8). Further, S6 Fig gathers IR and Raman plots for NBs∙∙∙Be_12_O_12_ complexes within configurations B and C.

From Fig 8 and S6 Fig, obvious alterations in the stretching bands of IR spectra for NBs were observed following the adsorption over Be_12_O_12_. In this regard, new peaks were found in the case of NBs∙∙∙Be_12_O_12_ complexes. In line with IR observations, significant changes were found in Raman spectra of NBs upon the adsorption over Be_12_O_12_. These observations affirmed that the Be_12_O_12_ would be a potent nanocarrier in the adsorption process of NBs.

### Recovery time

To systematically demonstrate the difficulty of NBs releasing from the surface of Be_12_O_12_, recovery time (*τ*) calculations were performed for all considered complexes. The calculated recovery time (*τ*) values for NBs∙∙∙Be_12_O_12_ complexes were compiled in Table 7.

**Table 7:** Calculated recovery time (*τ*, in *μ*s) values for NBs∙∙∙Be_12_O_12_ complexes within configurations A↔C.

Complex	Configuration	(*τ*)
**NB-C∙∙∙Be_12_O_12_**	A	2.78×10^3^
	B	3.27
	C	1.37×10
**NB-G∙∙∙Be_12_O_12_**	A	2.51×10^4^
	B	2.63×10
	C	1.22×10^2^
**NB-A∙∙∙Be_12_O_12_**	A	2.97×10^−3^
	B	4.22×10
	C	4.42
**NB-T∙∙∙Be_12_O_12_**	A	6.12×10^−1^
	B	1.18×10^−2^
	C	2.32×10^−6^
**NB-U∙∙∙Be_12_O_12_**	A	2.42×10^−1^
	B	4.17×10^−3^
	C	2.44×10^−5^

As found in Table 7, substantial *τ* values were obtained for all studied complexes with values ranging from 2.51×10^4^ to 2.32×10^−*6*^ *μ*s. Generally, as the adsorption energy increases, the recovery time (*τ*) becomes longer. For instance, recovery time (*τ*) values were found to be 2.42×10^−1^, 4.17×10^−3^, and 2.44×10^−5^ *μ*s for NB-U∙∙∙Be_12_O_12_ complexes within configurations A, B, and C, respectively.

## Conclusion

In the current study, a comprehensive DFT investigation was performed for the adsorption tendency of Be_12_O_12_ toward different NBs (i.e., NB-C, NB-G, NB-A, NB-T, and NB-U). According to ESP illustrations, nucleophilic sites were observed around NBs molecular surfaces with *V*_s,min_ values up to –63.8 kcal/mol for NB-C. Besides, obvious electrophilic regions were noticed above Be atoms with *V*_s,max_ = 146.1 kcal/mol. Concerning energy calculations, a favorable NBs-loading process over Be_12_O_12_ was confirmed through negative interaction (*E*_int_) and adsorption (*E*_ads_) values. Of all investigated complexes, configuration A had the most negative interaction energy (*E*_int_) value compared to other configurations. According to SAPT results, electrostatic (*E*_elst_) forces mainly dominated the total interaction energy, followed by induction (*E*_ind_) and dispersion (*E*_disp_) forces. From electronic attributes, the HOMO and LUMO isosurfaces of NBs systems were clearly dispersed as a result of adsorption over Be_12_O_12_. Notable changes were observed in DOS graphs of NBs following the adsorption over Be_12_O_12_. Exothermic and spontaneous natures were observed for all studied complexes from the obtained thermodynamic parameters. Suitable recovery time (*τ*) values were found for all studied complexes, ensuring the ability of the NBs to separate from the Be_12_O_12_. By the end, the obtained data assured the potential of Be_12_O_12_ to be a potent nanocarrier for NBs.

## Supporting information

S1 Fig Possible orientations of the NB-C∙∙∙Be_12_O_12_ complexes along with interaction energy (*E*_int_, kcal/mol) values. (DOCX)

S2 Fig QTAIM and 3D NCI diagrams for optimized NBs∙∙∙Be_12_O_12_ complexes within configurations B and C. (DOCX)

S3 Fig 2D NCI graphs for the optimized NBs∙∙∙Be_12_O_12_ complexes within configurations A, B, and C. (DOCX)

S4 Fig HOMO and LUMO plots for the optimized NBs∙∙∙Be_12_O_12_ complexes within configurations B and C. (DOCX)

S5 Fig DOS plots for the optimized NBs∙∙∙Be_12_O_12_ complexes within configurations B and C. (DOCX)

S6 Fig Infrared (IR) and Raman spectra for the optimized NBs∙∙∙Be_12_O_12_ complexes within configurations B and C. (DOCX)

S1 Table Cartesian atomic coordinates for the optimized structures. (DOCX)

S2 Table The route sections of the employed DFT calculations. (DOCX)

S1 Graphical abstract (TIF)
