# Supplementary material for: A DFT investigation on the potential of beryllium oxide (Be12O12) as a nanocarrier for nucleobases
Source: PLoS One. 2024 Nov 22;19(11):e0313885. doi: 10.1371/journal.pone.0313885 (PMC11584092; doi:10.1371/journal.pone.0313885)
Supplement: S1 Fig — (DOCX) [file pone.0313885.s001.docx]

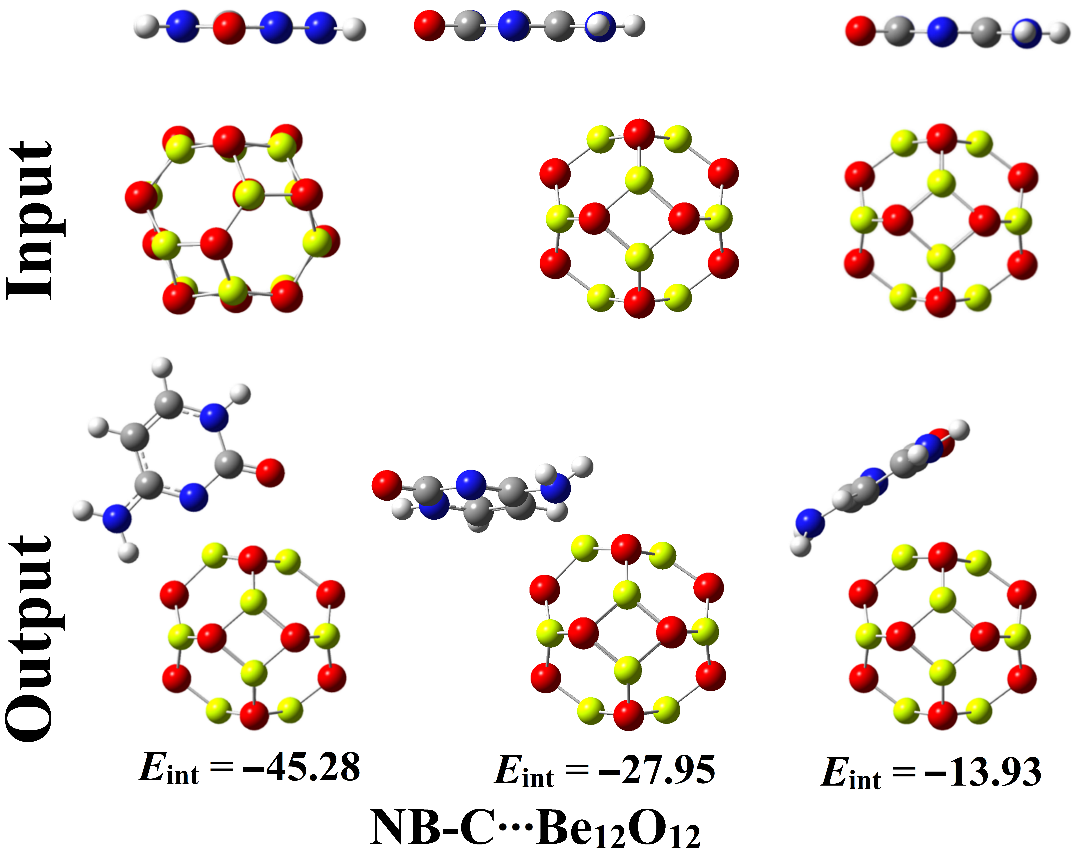


**S1 Fig.** Possible orientations of the NB-C∙∙∙Be_12_O_12_ complexes along with interaction energy (*E*_int_, kcal/mol) values.


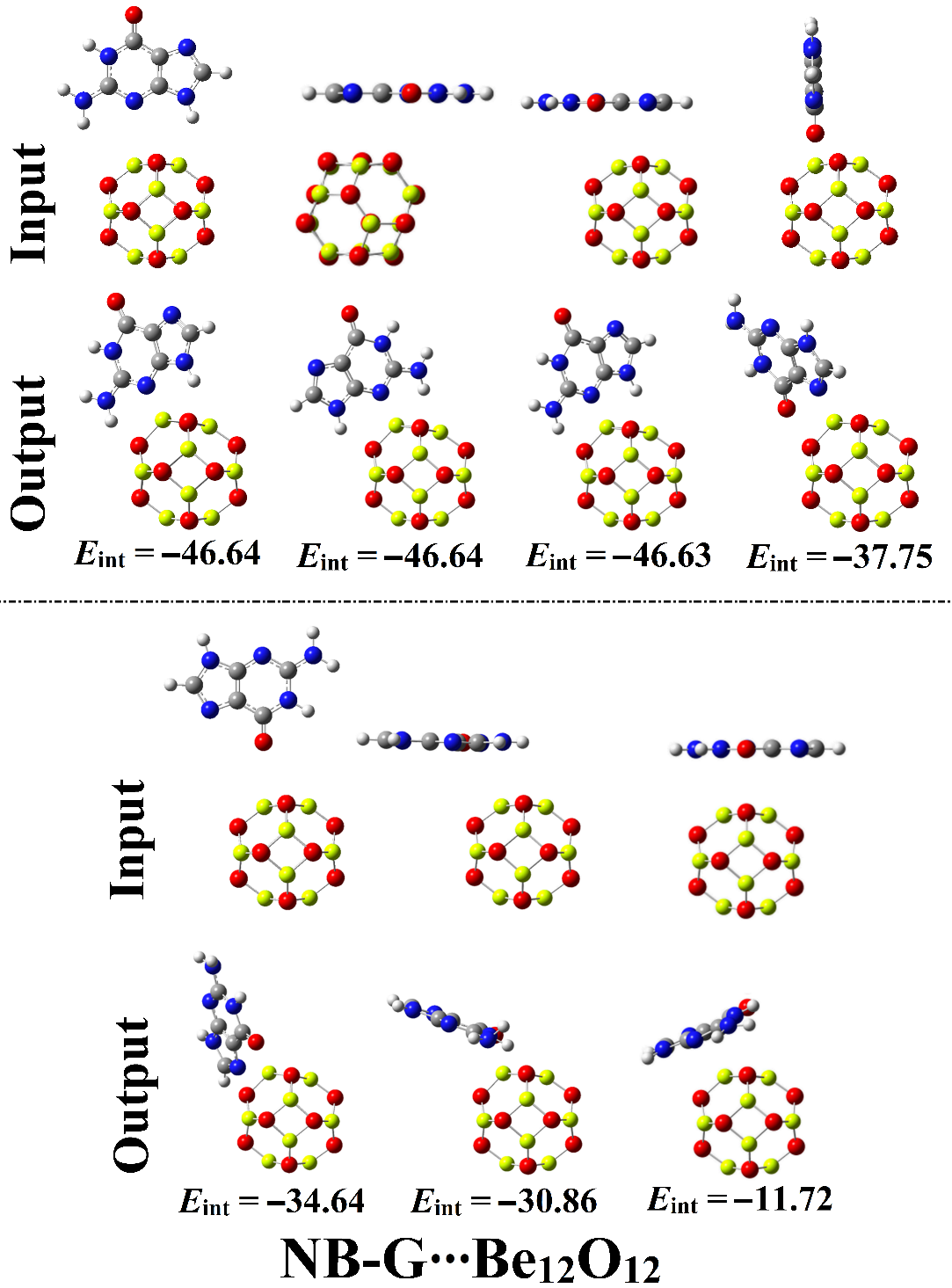


**S1 Fig.** (continued)

**
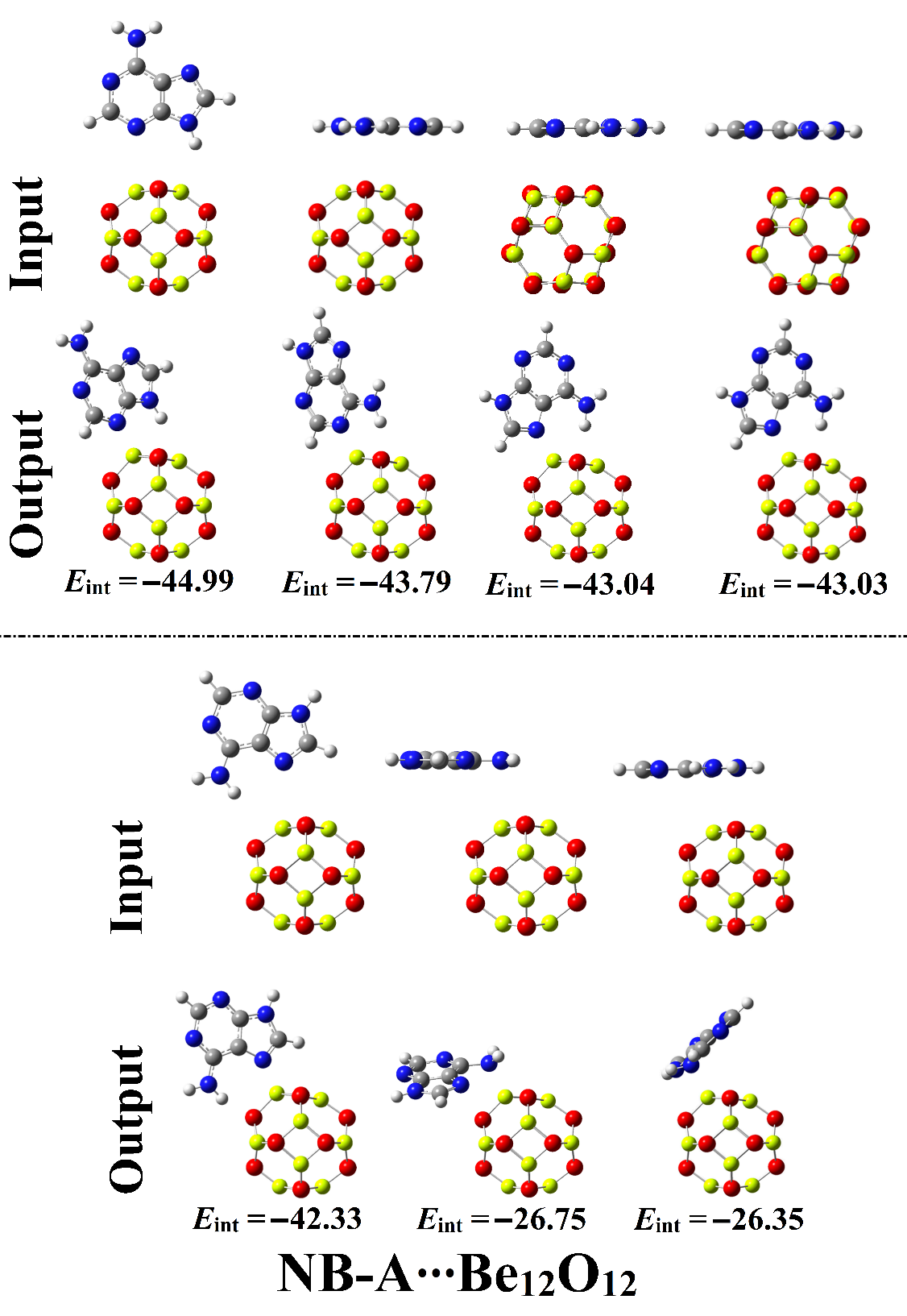
**

**S1 Fig.** (continued)


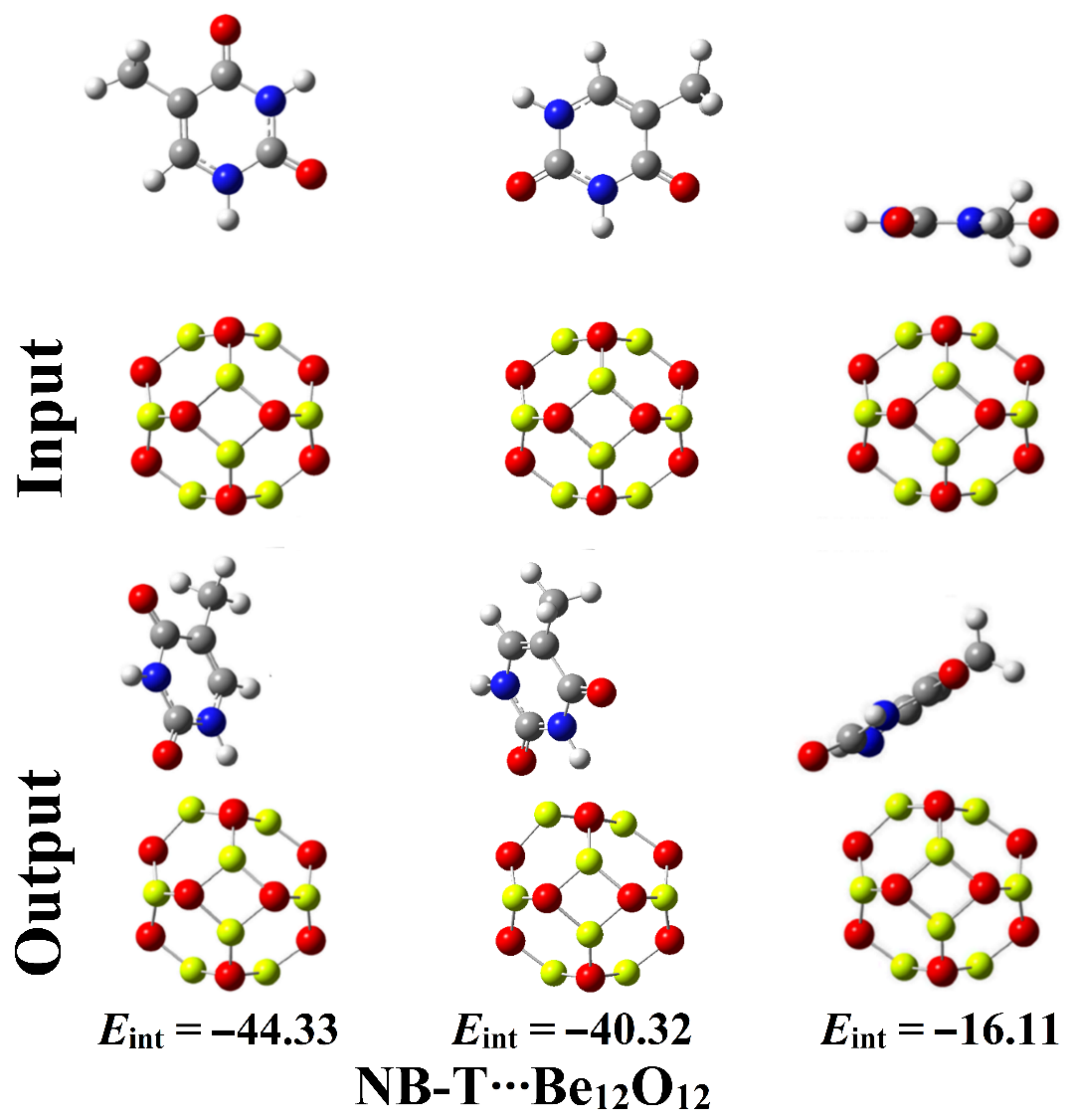


**S1 Fig.** (continued)


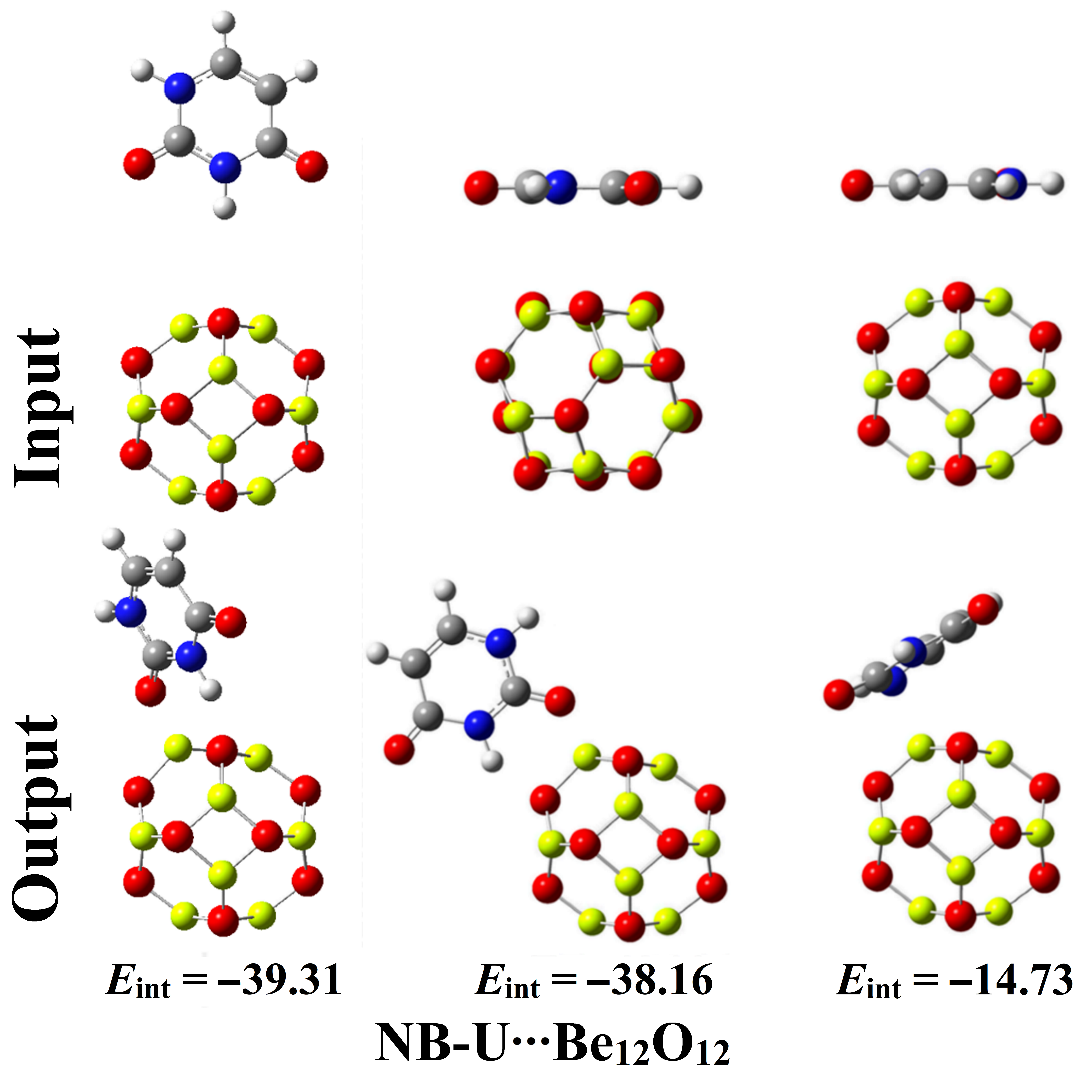


**S1 Fig.** (continued)
