# Supplementary figures and images for: A DFT investigation on the potential of beryllium oxide (Be12O12) as a nanocarrier for nucleobases
Source: PLoS One. 2024 Nov 22;19(11):e0313885. doi: 10.1371/journal.pone.0313885 (PMC11584092; doi:10.1371/journal.pone.0313885)

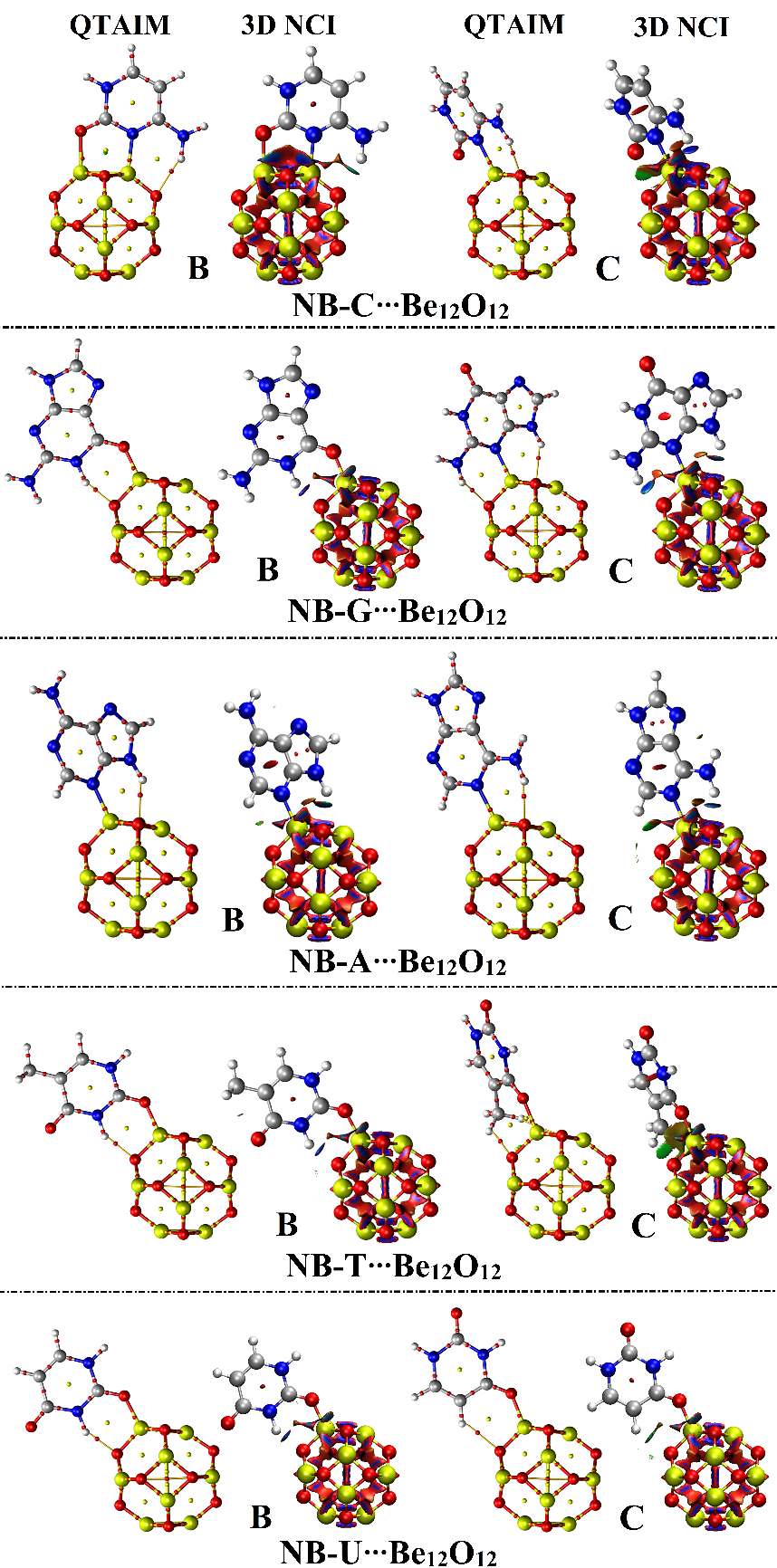


**S2 Fig.** QTAIM and 3D NCI diagrams for optimized NBs∙∙∙Be12O12 complexes within configurations B and C.

Supplement: S2 Fig — (DOCX) [file pone.0313885.s002.docx]

**
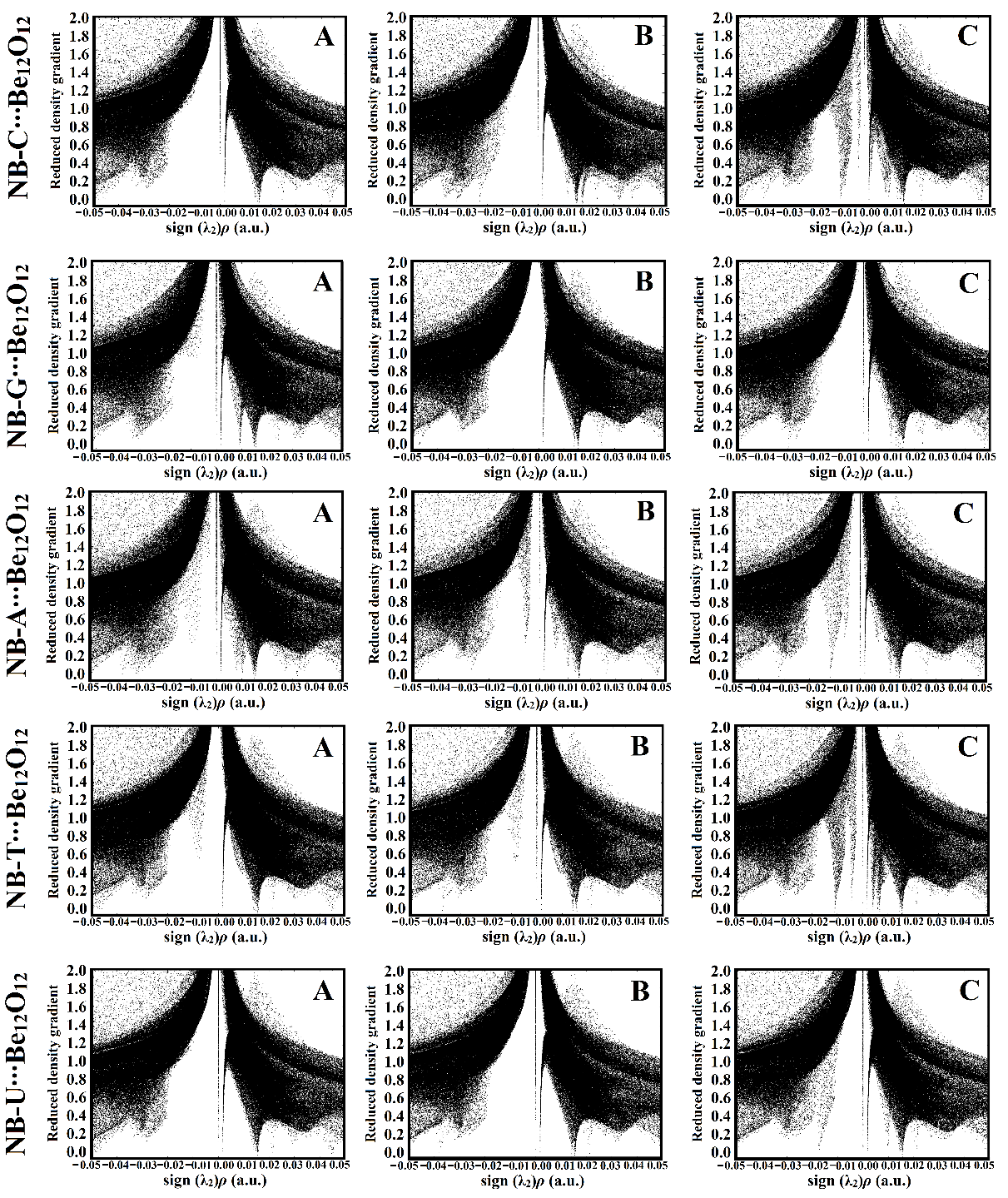
**

**S3 Fig.** 2D NCI graphs for the optimized NBs∙∙∙Be12O12 complexes within configurations A, B, and C.

Supplement: S3 Fig — (DOCX) [file pone.0313885.s003.docx]

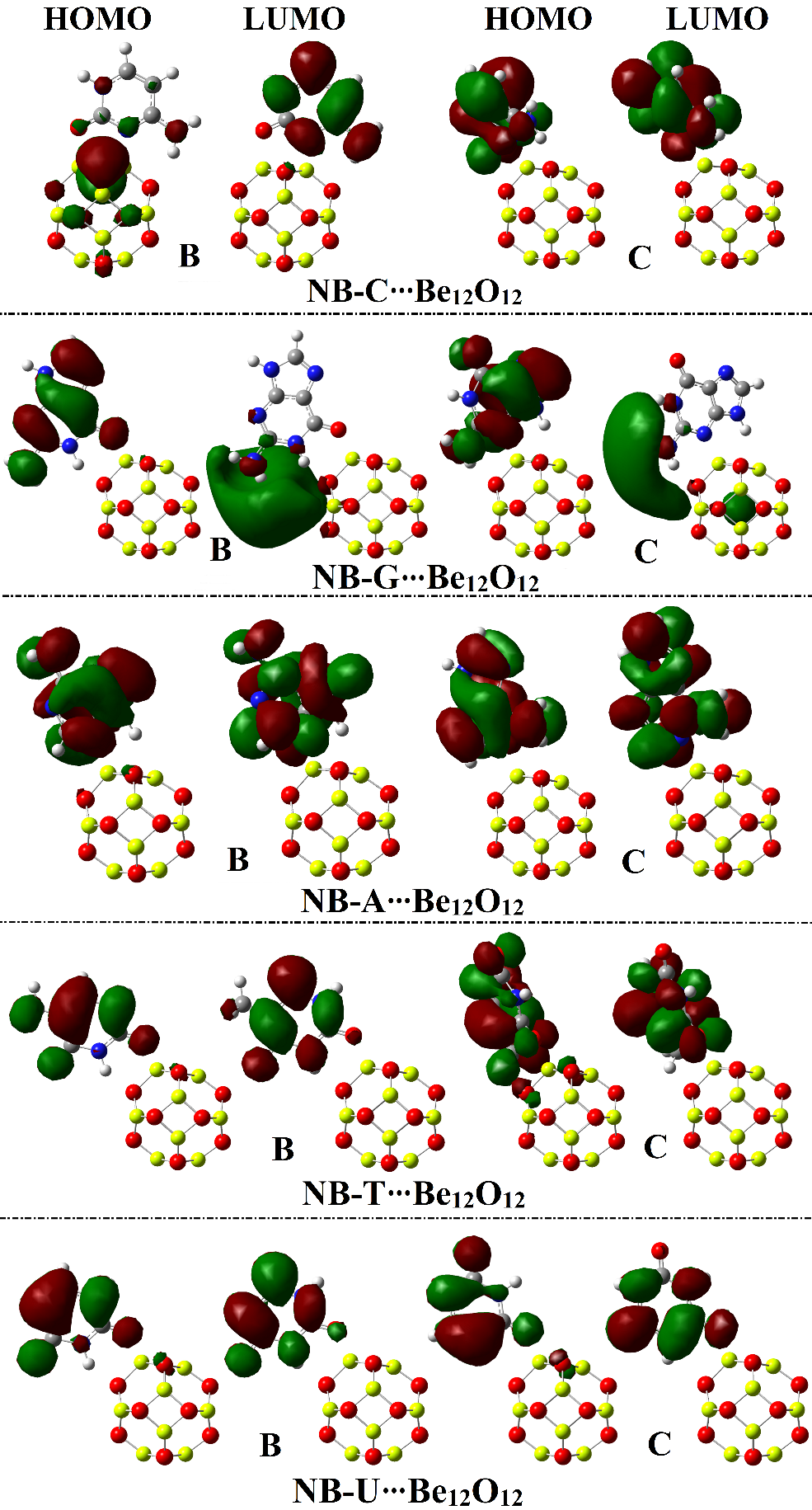


**S4 Fig.** HOMO and LUMO plots for the optimized NBs∙∙∙Be12O12 complexes within configurations B and C.

Supplement: S4 Fig — (DOCX) [file pone.0313885.s004.docx]

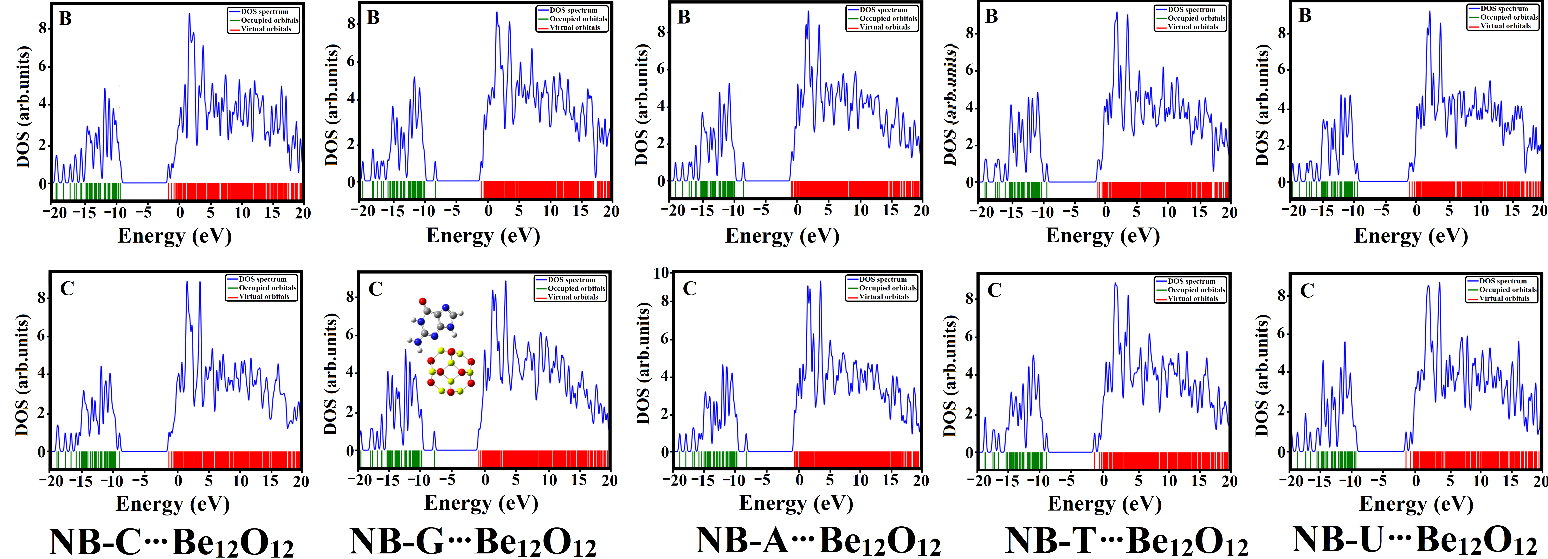


**S5 Fig.** DOS plots for the optimized NBs∙∙∙Be12O12 complexes within configurations B and C.

Supplement: S5 Fig — (DOCX) [file pone.0313885.s005.docx]

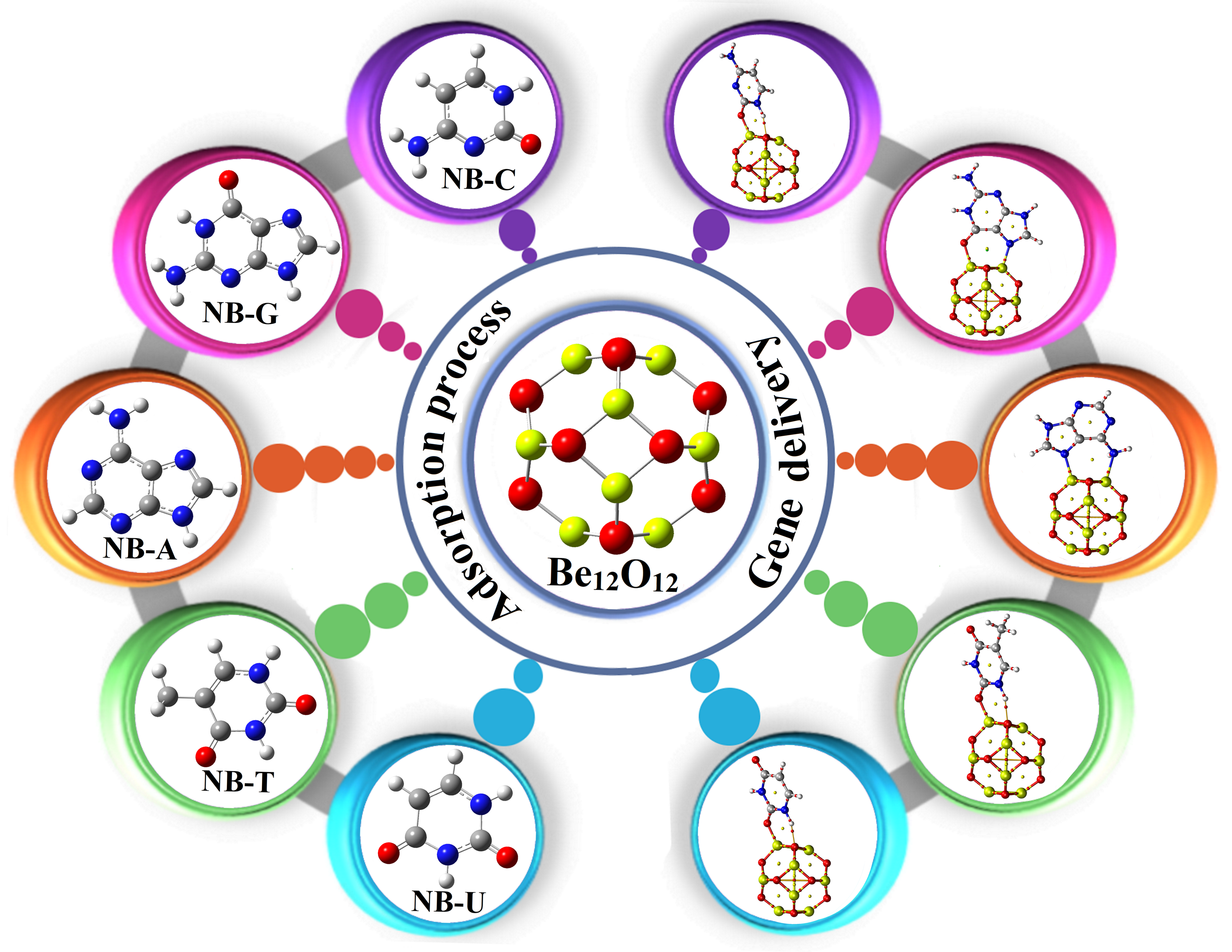

Supplement: S1 Graphical abstract — (TIF) [file pone.0313885.s009.tif]
