# Supplementary material for: A DFT investigation on the potential of beryllium oxide (Be12O12) as a nanocarrier for nucleobases
Source: PLoS One. 2024 Nov 22;19(11):e0313885. doi: 10.1371/journal.pone.0313885 (PMC11584092; doi:10.1371/journal.pone.0313885)
Supplement: S6 Fig — (DOCX) [file pone.0313885.s006.docx]

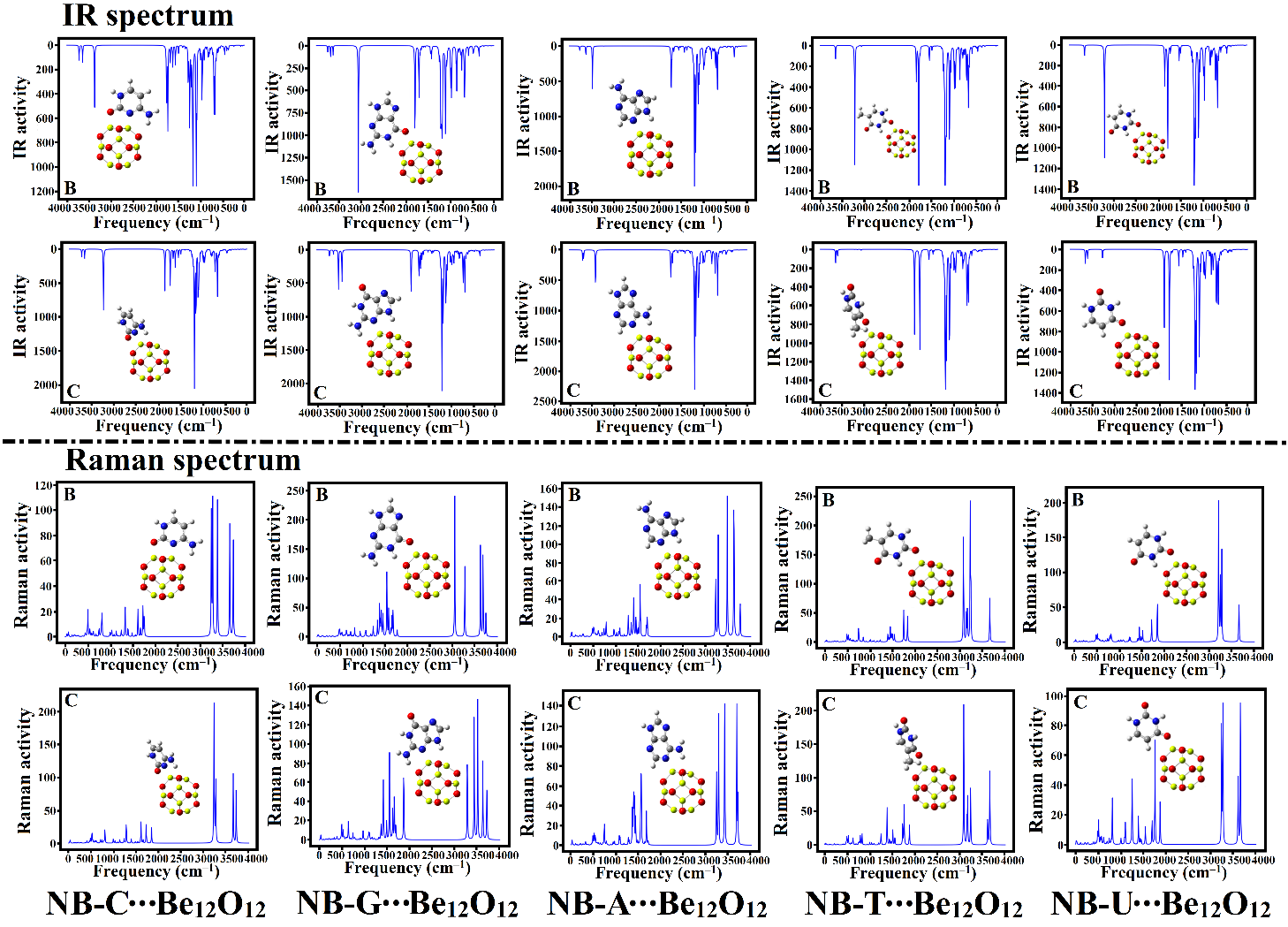


**S6 Fig.** Infrared (IR) and Raman spectra for the optimized NBs∙∙∙Be_12_O_12_ complexes within configurations B and C.
