# Supplementary material for: A DFT investigation on the potential of beryllium oxide (Be12O12) as a nanocarrier for nucleobases
Source: PLoS One. 2024 Nov 22;19(11):e0313885. doi: 10.1371/journal.pone.0313885 (PMC11584092; doi:10.1371/journal.pone.0313885)
Supplement: S1 Table — (DOCX) [file pone.0313885.s007.docx]

**S1 Table.** Cartesian atomic coordinates for the optimized structures

**Be_12_O_12_**

| **Cartesian atomic coordinates:**  O 3.16388900 -1.26192200 -1.16229400  O 3.15242700 1.47083100 -0.92922500  O 0.51107600 -1.81646700 -1.58716300  O 1.57606300 0.55305100 -2.47061900  O 0.56778000 2.34894200 -0.65794100  O -0.87368000 0.11000700 -1.31759800  O -0.57116500 1.27797100 1.14639800  O -0.55938000 -1.45649300 0.91331400  O 1.01683600 -0.53708100 2.45331200  O 2.08203000 1.83133700 1.57095700  O 2.02508900 -2.33387500 0.64179000  O 3.46708600 -0.09489100 1.30216700  Be 0.88509900 -1.76613300 1.47125300  Be 0.39266800 -0.34337600 -2.14445600  Be -0.58558700 -1.26386500 -0.59456700  Be 1.84310200 -2.08880200 -0.90759300  Be 3.19703400 -1.30031200 0.41605200  Be 2.89797800 0.12731300 -1.71859100  Be 2.20083300 0.35856900 2.12880600  Be 3.17845600 1.27865000 0.57837400  Be 0.74995600 2.10389500 0.89148900  Be -0.30493300 -0.11204500 1.70038000  Be -0.60466100 1.31592700 -0.43170400  Be 1.70812500 1.78118700 -1.48673500 |
| --- |

**Cytosine**

| **Cartesian atomic coordinates:**  C -0.65361700 -1.71769000 0.30351300  C -1.69210900 0.84961700 -0.17734900  C -2.53197900 -0.20259300 -0.06396300  C -0.28901800 0.55139100 -0.03617300  N -2.03993500 -1.44015400 0.16727500  N 0.19116300 -0.64685100 0.18824800  N 0.60177700 1.56516000 -0.14039400  H 0.31413800 2.51182300 -0.30973700  H 1.58000300 1.34810900 -0.03910400  O -0.31523900 -2.85948500 0.51070700  H -2.05348500 1.84989400 -0.36191100  H -3.60767700 -0.11175300 -0.14959900  H -2.64733300 -2.24170500 0.25617800 |
| --- |

**Guanine**

| **Cartesian atomic coordinates:**  C -2.67489300 0.91144000 0.28441300  C -0.55971100 0.86229000 0.37167600  C -0.93168300 -0.33482800 -0.21835000  C 0.84110600 1.12140900 0.58316600  C 1.11665900 -1.12248700 -0.48021200  N -2.29359600 -0.29289100 -0.27063300  N 1.60709600 0.01489600 0.09862700  N -1.66295500 1.62474200 0.67893100  N -0.15039500 -1.34840000 -0.67265600  N 2.03917300 -2.08001400 -0.82239800  O 1.40712100 2.06293900 1.07881800  H -3.71338900 1.19380500 0.36463100  H 2.59948200 0.09670100 0.27981700  H 2.95247200 -1.76651700 -1.11349100  H 1.65132900 -2.82239600 -1.38550100  H -2.88754700 -1.01549500 -0.64665800 |
| --- |

**Adenine**

| **Cartesian atomic coordinates:**  C -0.88166300 -1.46648700 0.25102900  C -3.79407200 1.67415600 -0.44028400  C -1.81171600 0.97614700 -0.20472600  C -2.61411100 -0.15088200 -0.06974200  C -0.42594700 0.76172900 -0.08506100  N -2.20392100 -1.40032200 0.15923800  N 0.01612200 -0.47734000 0.14421800  N -3.89431600 0.31922600 -0.22472500  N -2.56633200 2.10860500 -0.43569700  N 0.46111600 1.77097500 -0.19550800  H -0.46526900 -2.45121200 0.43652600  H -4.67008300 2.28639100 -0.59413100  H 0.14074800 2.70846600 -0.36845100  H 1.44256300 1.57111200 -0.10305900  H -4.73531800 -0.23514100 -0.18634800 |
| --- |

**Thymine**

| **Cartesian atomic coordinates:**  C -0.66191400 -1.66484700 0.33381000  C -1.62131200 0.99585900 -0.17404400  C -2.57845400 -0.11259800 -0.07371500  C -0.31615800 0.71861100 -0.02191400  N -2.00055900 -1.36376800 0.17404500  N 0.15359900 -0.55441900 0.22216600  O -0.24282100 -2.77590800 0.54795200  H -2.63748900 -2.14760100 0.24893900  O -3.77672000 -0.00049100 -0.18927500  H 1.13991200 -0.73386000 0.33258800  H 0.44624700 1.48572400 -0.08311800  C -2.17167000 2.36187600 -0.43985400  H -2.72815300 2.37283800 -1.37914000  H -2.87067000 2.65149000 0.34726100  H -1.37145100 3.10066600 -0.49311100 |
| --- |

**Uracil**

| **Cartesian atomic coordinates:**  C -0.75962200 -1.44960700 0.29473400  C -1.65742000 1.21027800 -0.21485000  C -2.64846300 0.14114100 -0.12437200  C -0.35618600 0.92316300 -0.05787500  N -2.09001800 -1.12389200 0.12724700  N 0.08521800 -0.35396300 0.18771000  O -0.35910100 -2.56562800 0.51168900  H -2.74090300 -1.89658400 0.19959100  O -3.84242400 0.26890500 -0.24505000  H 1.06642200 -0.55729300 0.30453400  H 0.41841500 1.67696300 -0.11530000  H -2.00606400 2.21265400 -0.40748500 |
| --- |
