# Supplementary material for: A DFT investigation on the potential of beryllium oxide (Be12O12) as a nanocarrier for nucleobases
Source: PLoS One. 2024 Nov 22;19(11):e0313885. doi: 10.1371/journal.pone.0313885 (PMC11584092; doi:10.1371/journal.pone.0313885)
Supplement: S2 Table — (DOCX) [file pone.0313885.s008.docx]

**S2 Table.** The route sections employed for DFT calculations.

| **Calculation** | **Route Section** |
| --- | --- |
| Optimization | opt M062X/6-311+G** geom=connectivity density=current int=ultrafine iop(6/7=1) scf=(xqc,verytight) |
| Frequency | # freq M062X/6-311+G** geom=connectivity density=current int=ultrafine iop(6/7=1) scf=(xqc,verytight) |
| MEP | # M062X/6-311+G** gfinput gfprint int=ultrafine iop(6/7=3) scf=(xqc,verytight) geom=connectivity cube=(medium,Potential) density=current  # M062X/6-311+G** nosymm gfinput gfprint int=ultrafine iop(6/7=3) scf=(xqc,verytight) geom=connectivity cube=(medium,Density) density=current |
| Energy | # M062X/6-311+G** geom=connectivity counterpoise=2 density=current int=ultrafine iop(6/7=1) scf=(xqc,verytight) |
| IR and Raman | # M062X/6-311+G** geom=connectivity freq=Raman scf=(xqc,verytight) iop(6/7=1) density=current int=ultrafine |
| wfn | # M062X/6-311+G** geom=connectivity pop=nbo gfinput gfprint int=ultrafine iop(6/7=3) scf=(xqc,verytight) Output=WFN density=current |
